# Multi-experiment assessment of soil nitrous oxide emissions in sugarcane

**DOI:** 10.1007/s10705-023-10321-w

**Published:** 2023-10-21

**Authors:** M. V. Galdos, J. R. Soares, K. S. Lourenço, P. Harris, M. Zeri, G. Cunha-Zeri, V. P. Vargas, I. A. M. Degaspari, H. Cantarella

**Affiliations:** 1 Rothamsted Research, Sustainable Soils and Crops, Harpenden, AL5 2JQ UK; 2 School of Agricultural Engineering (FEAGRI), University of Campinas (UNICAMP), Av. Cândido Rondon, 501, Campinas, SP 13083-875 Brazil; 3 Soils and Environmental Resources Centre, Agronomic Institute of Campinas (IAC), Av. Barao de Itapura 1481, Campinas, SP 13020-902 Brazil; 4 Rothamsted Research, Net Zero and Resilient Farming, North Wyke, Okehampton, Devon, EX20 2SB UK; 5 National Center for Monitoring and Early Warning of Natural Disasters (Cemaden), São José dos Campos, Brazil; 6 National Institute for Space Research (INPE), São José dos Campos, Brazil

**Keywords:** Nitrogen cycling, Emission factors, Mitigation, Bioenergy, Brazil

## Abstract

Soil nitrous oxide (N_2_O) fluxes comprise a significant part of the greenhouse gas emissions of agricultural products but are spatially and temporally variable, due to complex interactions between climate, soil and management variables. This study aimed to identify the main factors that affect N_2_O emissions under sugarcane, using a multi-site database from field experiments. Greenhouse gas fluxes, soil, climate, and management data were obtained from 13 field trials spanning the 2011–2017 period. We conducted exploratory, descriptive and inferential data analyses in experiments with varying fertiliser and stillage (vinasse) type and rate, and crop residue rates. The most relevant period of high N_2_O fluxes was the first 46 days after fertiliser application. The results indicate a strong positive correlation of cumulative N_2_O with nitrogen (N) fertiliser rate, soil fungi community (18S rRNA gene), soil ammonium (NH_4_^+^) and nitrate (NO_3_^−^); and a moderate negative correlation with amoA genes of ammonia-oxidising archaea (AOA) and soil organic matter content. The regression analysis revealed that easily routinely measured climate and management-related variables explained over 50% of the variation in cumulative N_2_O emissions, and that additional soil chemical and physical parameters improved the regression fit with an *R*^2^ = 0.65. Cross-wavelet analysis indicated significant correlations of N_2_O fluxes with rainfall and air temperature up to 64 days, associated with temporal lags of 2 to 4 days in some experiments, and presenting a good environmental control over fluxes in general. The nitrogen fertiliser mean emission factors ranged from 0.03 to 1.17% of N applied, with urea and ammonium nitrate plus vinasse producing high emissions, while ammonium sulphate, ammonium nitrate without vinasse, calcium nitrate, and mitigation alternatives (nitrification inhibitors and timing of vinasse application) producing low N_2_O-EFs. Measurements from multiple sites spanning several cropping seasons were useful for exploring the influence of environmental and management-related variables on soil N_2_O emissions in sugarcane production, providing support for global warming mitigation strategies, nitrogen management policies, and increased agricultural input efficiency.

**Supplementary Information:**

The online version contains supplementary material available at 10.1007/s10705-023-10321-w.

## Introduction

Global human-induced nitrous oxide (N_2_O) emissions have increased by 30% over the past four decades, mostly from nitrogen fertiliser application on cropland (Tian et al. 2020). Brazil and other emerging economies are responsible for a large share of these increased emissions. Sugarcane is an important crop globally, with close to 26.8 Mha harvested in over 100 countries, with Brazil accounting for over a third of the harvested area in 2020 (FAO 2022). Besides being used for centuries as a source of sugar, the crop has an important role as a bioenergy feedstock. Renewable sources represent 45% of the Brazilian energy matrix, with sugarcane ethanol and bagasse corresponding to 39% (MME 2019).

In Brazil, sugarcane-derived ethanol use for transportation emits 82% less GHGs compared to petrol use (Jaiswal et al. 2017). In-field GHG emissions have progressively decreased in recent years with the phase-out of pre-harvest burning of sugarcane in Brazil, with the potential benefit of increased soil carbon sequestration from the decomposition of crop residues (Galdos et al. 2009, 2010). With the reduction of biomass burning and other sources of GHG, N_2_O emissions from fertiliser application, when converted to CO_2_-equivalent, have become increasingly important in the carbon footprint of sugarcane products.

Soil N_2_O emissions are highly variable due to factors such as climate conditions, soil properties, and management practices. The N_2_O emission factor (EF) default value for national inventories was recently updated in the guidelines of the Intergovernmental Panel on Climate Change (IPCC), e.g. EF of synthetic fertiliser (Tier 1) was 1.0% (0.3–3.0%) of N applied (IPCC 2006), and changed to disaggregated values of 0.5% (0.0–1.1%) in dry climate, and 1.6% (1.3–1.9%) in wet conditions (IPCC 2019). The average N_2_O-EF reported for sugarcane fields was 1.2% (1.0–1.5%) in a global study (Yang et al. 2021) and 0.7% (0.1–3.0%) in Brazil (Carvalho et al. 2021), which is fundamental to evaluating the environmental impact of ethanol.

Using an IPCC N_2_O-EF of 1.0%, Carvalho et al. (2021) demonstrated that the N_2_O emission can account for roughly half of the total GHG emission in bioethanol production. However, compared with IPCC values, the use of regional data (0.7%) reduced the total GHG emissions by 17, 18, and 21% when the fertilisers were ammonium nitrate (AN), urea, and ammonium sulphate (AS), respectively. A significant reduction in GHG emissions by choosing ethanol instead of petrol was evaluated considering N_2_O-EF of 1% (Cavalett et al. 2017), while N_2_O-EF as high as 5% could negate the carbon offsetting benefits of biofuels (Crutzen et al. 2007).

Site-specific crop and soil management conditions can lead to distinct results in total N_2_O emissions in sugarcane fields. Gonzaga et al. (2018) observed higher N_2_O-EF from N fertiliser by increasing straw levels from 0 to 15 Mg ha^−1^ (roughly 1 Mg ha^−1^ is equal to 4.0–4.5 kg N ha^−1^; Lourenco et al. 2018), but Vasconcelos et al. (2022) showed no effect of straw levels, and Pitombo et al. (2017) reported reduction in N_2_O-EF in soil covered with straw. Soares et al. (2016) reported a 95% reduction in N_2_O emission from urea adding nitrification inhibitors (Dicyandiamide-DCD and 3,4-dimethylpyrazole phosphate-DMPP), while Wang et al. (2016) showed a reduction of less than 36%. Combining application of nitrogen (N) fertiliser with stillage resulting from ethanol production (vinasse: 0.5–3.0 g N L^−1^) can lead to threefold increases in N_2_O emissions. In a study conducted by Lourenço et al. (2019), the N_2_O-EF from ammonium nitrate increased from 0.23% to 0.94% of N applied, and it reached 3% of N applied, when vinasse was applied at the same time as N fertilisers (Carmo et al. (2013).

Understanding how N_2_O fluxes are correlated with management and environmental conditions can help design strategies to mitigate emissions in sugarcane production. Soares et al. (2016) showed via a multiple linear regression (*R*^2^ = 0.47), N_2_O emissions correlating with the abundance of ammonia oxidising bacteria (AOB), precipitation, soil NH_4_^+^–N, NO_3_^−^–N, pH and CO_2_ emission due to N fertilisation in sugarcane in Brazil. Furthermore, Lourenço et al. (2018, 2022) expand the list of variables affecting N_2_O fluxes in sugarcane systems with straw and vinasse application, including factors such as bacterial genes linked to denitrification, ammonia oxidising archaea, fungal denitrifiers, air and soil temperature, and water-filled pore space.

Grouping individual studies can summarise the factors that may affect N_2_O emission in sugarcane fields. In a meta-analysis, Yang et al. (2021) showed higher cumulative N_2_O emissions when synthetic fertiliser was applied with organic amendments. On the other hand, no effect was observed in N_2_O-EF from N fertilisers due to the presence of straw (Abalos et al. 2022). Moreover, other management strategies that may influence N_2_O emissions from sugarcane fields, such as N sources, vinasse, and microbial activity, have yet to be investigated.

Therefore, management practices in sugarcane can affect the N_2_O emissions differently, and a better understanding of the factors influencing them can help in prediction and mitigation. To our knowledge, only one meta-analysis study has been published for N_2_O emissions in sugarcane (Yang et al. 2021), meaning our study with its rich datasets provides further analytical opportunities to understand such emissions, and fills a knowledge gap where little is known about the temporal influences on N_2_O fluxes. Thus, the aim of the present study was to identify the main factors that affect N_2_O emissions in sugarcane production, using a unique database containing daily N_2_O fluxes measured with a standard protocol in a variety of soil, climate, and management conditions.

## Material and methods

### Study sites and database

Flux measurements of N_2_O were obtained from 13 sugarcane trials conducted in the 2011–2017 period, at three experimental stations located in the main sugarcane-growing region in Brazil (Figure S1). The experiments involved management practices related to N fertilisation and organic amendments, such as various N fertiliser types and rates, vinasse application and post-harvest straw management (Table 1), which reflect both current management systems and mitigation alternatives (nitrification inhibitors and timing of vinasse application). All trials followed a similar experimental design, with each plot consisting of five rows of sugarcane spaced 1.5 m apart along a 10 m length, arranged into four blocks, each containing one or two chambers for gas sampling. The datasets were derived from a network of experiments conducted by researchers from the Agronomic Institute of Campinas (IAC) using the same protocol (Vargas 2013; Soares et al. 2015, 2016; Lourenço et al. 2018, 2019; Degaspari et al. 2020). The soils in the areas were classified as Red Latosol and Nitisol (Embrapa 2006).

**Table 1:** Description of trials, including location, crop stage, sugarcane variety, nitrogen fertiliser type and rate, vinasse rate and timing of application, and amount of post-harvest sugarcane straw

Location	Trial	Sugarcane crop		N fertiliser		Straw	Vinasse		References
		Stage	Cultivar	Type	kg N ha^−1^	Mg ha^−1^	m^3^ ha^−1^	Timing	
Santa Elisa Experimental Station, Campinas, São Paulo	C1	R1	SP791011	UR, UR + DCD, UR + DMPP, PSCU	0, 120	0	–	–	Soares et al. (2015)
	C2	R2	SP791011	UR, UR + DCD, UR + DMPP, PSCU	0, 120	0	–	–	Soares et al. (2015)
	C3	R3	SP791011	UR, UR + DCD, UR + DMPP, PSCU, CN	0, 120	0	–	–	Soares et al. (2016)
APTA station, Piracicaba, São Paulo	P1	P	IAC95-5000	UR, CAN	0, 30, 60, 90	0	–	–	Degaspari et al. (2020)
	P2	R2	IAC95-5000	UR, CAN	0, 60, 120, 180	14	–		Degaspari et al. (2020)
	P3	R3	IAC95-5000	UR, CAN	0, 60, 120, 180	14	–	–	Degaspari et al. (2020)
	P4	R1	SP 81-3250	AS, AS + I	0, 100, 150	14	–	–	Vargas (2013)
	P5	R2	SP 81-3250	AN, AN + I	0, 50, 100, 150	14	–	–	Vargas (2013)
	P6	R2	RB86-7515	AN	0, 100	9	0, 17*, 100	V0, VN0, V + N, V,N	Lourenço et al. (2018, 2019)
	P7	R3	RB86-7515	AN	0, 100	12	0, 17*, 100	V0, VN0, V + N, V/N	Lourenço et al. (2018, 2019)
	P8	R4	RB86-7515	AN	0, 100	16	0, 17*, 100	V0, VN0, V + N, N/V	Lourenço et al. (2018, 2019)
APTA station, Jaú, São Paulo	J1	R1	SP 81-3250	AS, AS + I	0, 100, 150	14	–	–	Vargas (2013)
	J2	R2	SP 81-3250	AN, AN + I	0, 50, 100, 150	14	–	–	Vargas (2013)

P, Plant; R, Ratoon (1,2,3 years); UR, Urea; UR + DCD, Urea with Dicyandiamide nitrification inhibitor; UR + DMPP, Urea with 3,4-Dimethylpyrazole Phosphate nitrification inhibitor; PSCU, Polymer sulphur coated urea; CN, Calcium nitrate, CAN, Calcium ammonium nitrate, AS, Ammonium sulphate; AN, Ammonium nitrate; V0, no vinasse applied; VN0, vinasse with no N fertiliser; V + N, vinasse applied with N; V/N, Vinasse applied 30 days before N; N/V, Vinasse applied 30 days after N.*rate for concentrated vinasse

Key variables for the soil, plant and atmosphere interface were measured in the trials, including GHG fluxes, climate data, stalk yields, crop residue (straw) rates, and soil chemical, physical, and biological properties (Table 2). Besides standard agronomic soil variables, the dataset includes abundances of the functional genes (archaeal and bacterial *amoA*, bacterial and fungal *nirK*, and bacterial *nirS* and *nosZ)*, which encode proteins involved in nitrification and denitrification processes, and ribosomal RNA genes indicating total bacteria abundance (16S rRNA) and total fungi abundance (18S rRNA). The climate variables were obtained from weather stations located near the field plots (Ciiagro 2020).

**Table 2:** Variables measured and units in the sugarcane trials included in the dataset

Variables	Description	Unit	Trials
N_2_O	Nitrous oxide fluxes	mg N m^−2^ day^−1^	All
CH_4_	Methane fluxes	mg C m^−2^ day^−1^	All
CO_2_	Carbon dioxide fluxes	g C m^−2^ day^−1^	All
N_2_O cumulative	Cumulative nitrous oxide emission	mg N m^−2^	All
N_2_O-EF	Emission factor	% of N: applied	All
N_2_O intensity	Emission intensity	g N_2_O Mg^−1^ stalk	P1-8, J1-2
CH_4_	Cumulative methane emission	mg C m^−2^	All
CO_2_	Cumulative carbon dioxide emission	g C m^−2^	All
Date	Day of the year	dd/mm/yy	All
DAF	Days after fertiliser application	day	All
N rate	Fertiliser-N applied	kg ha^−1^	All
Straw rate	Straw left on soil after harvest	kg ha^−1^	P2–P8
Vinasse	Stillage applied	kg ha^−1^	P6–P8
N source	Fertilisers and vinasse	kg ha^−1^	All
NO_3_^−^	Nitrate content	mg N kg^−1^ soil	All
NH_4_^+^	Ammonium content	mg N kg^−1^ soil	All
Precipitation	Daily precipitation	Mm	All
Tmax	Daily maximum temperature	°C	All
Tmin	Daily minimum temperature	°C	All
Tmean	Daily mean temperature	°C	All
Air Temp	Air temp. at GHG sampling time	°C	All
WFPS	Water filled pore space	%	All
Sand	Sand content	%	All
Silt	Silt content	%	All
Clay	Clay content	%	All
BD	Bulk density	g cm^−3^	All
OM	Organic matter content	g dm^−3^	All
pH	pH in CaCl_2_	–	All
K	Potassium	mmol_c_ dm^−3^	All
Ca	Calcium	mmol_c_ dm^−3^	All
Mg	Magnesium	mmol_c_ dm^−3^	All
P	Phosphorus	mg dm^−3^	All
Yield	Sugarcane stalk yield	Mg stalk ha^−1^	P1-8, J1-2
Soil Temp	Soil temp. at GHG sampling time	°C	C1-3, P1-3, P6-8, J1
CEC	Cation exchange capacity	mmol_c_ dm^−3^	C1-3, P1-5, J1-2
Cu	Copper	mg dm^−3^	C1-3, P4-5, J1-2
Fe	Iron	mg dm^−3^	C1-3, P4-5, J1-2
Mn	Manganese	mg dm^−3^	C1-3, P4-5, J1-2
Zn	Zinc	mg dm^−3^	C1-3, P4-5, J1-2
V	Base saturation	%	P1-8, J1-2
H + Al	H^+^ + Al^+3^, potential acidity	mmol_c_ dm^−3^	P6-8
AOA	*amoA*-AOA	Gene copies g^−1^ soil	C1-3, P6-8
AOB	*amoA*-AOB	Gene copies g^−1^ soil	C1-3, P6-8
*nirK*	*nirK*	Gene copies g^−1^ soil	C1-3, P6-8
*nirS*	*nirS*	Gene copies g^−1^ soil	C1-3, P6-8
*nosZ*	*nosZ*	Gene copies g^−1^ soil	C1-3, P6-8
16S	16S rDNA	Gene copies g^−1^ soil	C1-3, P6-8
*nirK*-Fungi	*nirK*-Fungi	Gene copies g^−1^ soil	P6-8
18S	18S rDNA	Gene copies g^−1^ soil	P6-8

N_2_O cumulative emission per season (~ 330 d). Vinasse: 25.7–69.7 g C L^−1^, 0.5–3.0 g N L^−1^. Straw: 450 g C kg^−1^, 4.0–4.5 g N kg^−1^

GHG fluxes (CO_2_, CH_4_ and N_2_O) from all experiments were measured by cylindrical static chambers with 0.2 m in height and 0.3 m in diameter, inserted at a 0.05 m soil depth. Chambers were positioned in the inter-row (0.75 m from the sugarcane row) and partially in-row (0.10 m from the sugarcane row), to account for spatial differences in nitrogen fertilizer band application. Gases were sampled in the early morning three times per week during the first three months after fertiliser application, then biweekly. Three or four gas samples were collected in the 30 min following closure of the chambers, in either three (0–15–30 min) or four (0–10–20–30 min) measurements. After sampling, the gases were immediately stored in pre-evacuated Extainers® vials (Labco Limited, Ceredigion, United Kingdom) and analysed on a Shimadzu gas chromatograph (GC-2014). Although all three gases (CO_2_, CH_4_, and N_2_O) were analysed, this study focussed on N_2_O fluxes as a key component of the environmental footprint of sugarcane production.

The N_2_O flux was calculated by linear interpolation of the three or four sampling times (0–10–20–30 min), obtaining the angular coefficient. The cumulative N_2_O emission was calculated by linear interpolation between adjacent sampling dates. More details can be found in the studies used (Vargas 2013; Soares et al. 2015, 2016; Lourenço et al. 2018, 2019; Degaspari et al. 2020).

### Descriptive, exploratory and inferential analyses

#### Overview

Descriptive, exploratory and inferential statistical analyses were undertaken to characterise the main factors that affect N_2_O emissions in sugarcane production. The original data from each trial was processed into a standard format for inclusion in the study database. Quality assessment included unit conversion, range tests, and visual inspection of homogeneity. The complete dataset was comprised of 49 quantitative and categorical variables from 13 trials encompassing a period of six years, with a total of close to 100,000 unique data points. When necessary, variables were transformed to deal with heavily skewed data and to promote robust analyses. For regression-type models, collinearity effects were catered for either by removal of strongly collinear data or within the model itself through some penalised term (e.g. a ridge term). Compositional data were handled simply by removing one class (e.g. remove clay say, from sand/silt/clay soil texture data). In the first instance, measurements for N_2_O and N_2_O-EFs were described, summarised, and visualised using simple boxplots, coupled with ANOVAs and related analyses.

Global and local temporal co-dependencies between N_2_O and rainfall and air temperature were explored via a series of cross-wavelet analyses. This analysis provided information on peak N_2_O fluxes given changes in the weather. Next, and similarly exploratory, a regression tree (RT) analysis was conducted to see how management, climate, and soil conditions influence N_2_O fluxes. Using a data subset directed by the RT analysis, correlation analyses were conducted to explore paired relationships and trends in the N_2_O processes and further highlight any collinear effects among the N_2_O predictor variables. Finally, an inferential multivariate analysis using linear regression was performed with N_2_O as the response, and whose coefficients were estimated using ordinary least squares (OLS) and assessed for significance from zero.

All analyses were conducted using R software, version 3.6.1 (R core team 2021), aside from the cross-wavelet analysis which was conducted using Python 3.6 with scripts from the PyCWT package (https://pypi.org/project/pycwt/). A detailed description of the statistical analyses can be found in Supplementary Material.

#### Differences in emission factors and emission intensity

The emission factor (EF) was calculated considering the cumulative N_2_O and N applied in the chambers for each treatment:

1 $${\text{EF}} = \frac{{\left( {{\text{N}}_{2} O_{{{\text{treat}}}} - {\text{N}}_{2} {\text{O}}_{{{\text{control}}}} } \right)}}{{N \,{\text{applied}}}}*100$$

where EF is N_2_O emission factor (% of N applied); N_2_O treat (mg N m^−2^) and N_2_O control (mg N m^−2^) are the cumulative emissions of the fertilised and unfertilized chambers, respectively; and N applied is the amount of N (mg N m^−2^) added to the chamber as synthetic fertiliser and/or vinasse. The N_2_O emission intensity was calculated by dividing the cumulative N_2_O emissions in the season by the fresh weight yield of sugarcane stalks harvested.

The N_2_O-EFs were conditionally analysed according to the use of conventional fertilisers and for different mitigation treatments (nitrification inhibitor and timing of vinasse application). Firstly, this involved the simple presentation of conditional summary statistics (means, medians, standard deviations, IQRs, etc.) and boxplots. Secondly, formal analyses were conducted, using ANOVAs and Kruskal–Wallis (KW) rank sum tests (Vargha and Delaney 1998) in order to test whether EF variability was significantly different between conventional fertilisers and mitigation treatments. The ANOVAs and KW tests were supplemented by their respective post-hoc analysis (Tukey Honest Significant Differences—HSD) (Tukey, 1949) and the Dunn test (Dunn 1964) in order to determine which fertiliser or which mitigation had significantly different EF distributions (as an ANOVA or KW test only indicates at least one ‘category’ is different, but not which ‘category’). KW/Dunn tests represent robust alternatives to ANOVA/HSD tests, where the latter is resistant to outlying EFs (i.e., the different tests are analogous to the presentation of median/IQRs as well as means/SDs).

## Results

### Differences in emission factors and emission intensity

Daily N_2_O fluxes had a period of high emissions after fertiliser application, which was similar in all experiments and reached almost 150 mg N m^−2^ day^−1^ in trial C3 (Figure S2—top panel). The events of high emissions decrease until 100 DAF, showing low values afterwards with rare exceptions (Figure S2—middle panel). Some differences can be observed between trials with conditional boxplots, e.g., trial P2 had higher cumulative N_2_O emissions, which corresponded to a median of 710 mg N m^−2^, while J1 resulted in the lowest values, a median lower than 40 mg N m^−2^ (Figure S2—bottom panel).

**Fig. 1:**
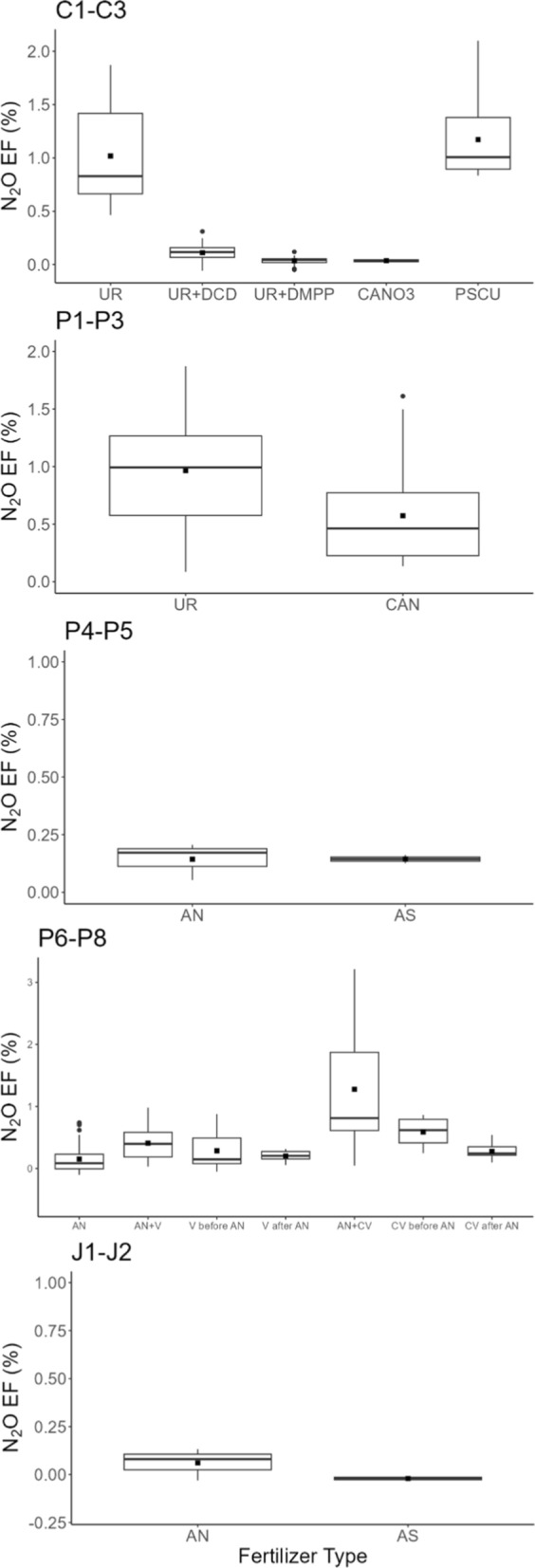
Nitrous oxide emission factors (% of N fertiliser applied) according to N source in 13 sugarcane trials. AN: Ammonium Nitrate; AS: Ammonium Sulphate; CAN: Calcium Ammonium Nitrate; CN: Calcium Nitrate; PSCU: polymer sulphur -coated urea; UR: Urea; UR + DCD: Urea with Dicyandiamide; UR + DMPP: Urea with 3,4-Dimethylpyrazole Phosphate; V: Vinasse; CV: Concentrated vinasse

Via the ANOVAs and associated tests, significant differences in N_2_O-EF between N sources were observed for each experiment group (Tables S1 and S2). In trials C1–C3—in which fertilisers were applied at 120 kg N ha^−1^ -UR and PSCU resulted in higher N_2_O-EF, with mean values of 1.0% and 1.2% of N applied, compared to UR containing nitrification inhibitors (DMPP and DCD) and calcium nitrate, which resulted in N_2_O-EF lower than 0.1%, as depicted in the conditional boxplots (Fig. 1). The UR treatment resulted in higher mean N_2_O-EF (1.0%) than CAN (0.6%) in P1–P3, with fertiliser rates ranging from 30 to 180 kg N ha^−1^. In P4-P5 and J1–J2—with fertiliser rates in the 50–150 kg N ha^−1^ range, the mean N_2_O-EFs were lower than 0.2% of N applied as AN and AS (Fig. 1), showing no statistically significant differences via the ANOVAs and associated tests (Table S1 and S2). In P6-P8 (with a uniform rate of 100 kg N ha^−1^), the application of AN with concentrated vinasse resulted in a mean N_2_O-EF of 1.3% of N applied (Fig. 1), where changing the time of vinasse application significantly decreased those emissions to 0.6% when vinasse was applied one month before AN, and to 0.3% when concentrated vinasse was applied one month after AN (see Tables S1 and S2).

Grouping the same N source from different trials, the mean N_2_O-EFs ranged from 0.03 to 1.17% of N applied (Table 3). The highest N_2_O-EFs were UR and PSCU, with mean values of 0.98% and 1.17%, respectively. Mean EFs for CAN (0.57%) and AN (0.50%) were higher than CN, AS, and UR + Nitrification Inhibitors (NIs; DCD and DMPP), with N_2_O-EFs of 0.1% of N applied or lower (Table 3). A weighted average of EFs considering the number of treatments in each fertiliser type, excluding the mitigation treatments (UR + DCD and UR + DMPP) and the coated urea treatment (PSCU), amounts to a mean EF of 0.6 (± 0.03)%. The intensity of N_2_O emission ranged from 2 to 150 g N_2_O per Mg of sugarcane stalk yield (Figure S3). The N_2_O intensity results were similar to those with N_2_O-EF, with higher values with UR and AN combined with concentrated vinasse. The N rate increased N_2_O intensity for UR, AN, AS, and CAN (Figure S3).

**Table 3:** Summary statistics for N_2_O emission factors (EF, %) conditional to conventional fertilisers, mitigation treatments, and coated urea in the experiment trials

Fertiliser type	Mean	median	SD	SEM	IQR	max	min	n
AN^*^	0.50	0.29	0.62	0.00	0.53	3.21	− 0.10	270
AS	0.06	0.06	0.10	0.02	0.15	0.16	− 0.03	4
CAN	0.57	0.46	0.40	0.01	0.55	1.61	0.13	36
CN	0.04	0.03	0.01	0.00	0.02	0.05	0.02	4
UR	0.98	0.92	0.47	0.01	0.68	1.87	0.09	48
UR + DCD	0.11	0.12	0.09	0.00	0.09	0.31	− 0.06	20
UR + DMPP	0.03	0.04	0.04	0.00	0.04	0.12	− 0.05	20
PSCU	1.17	1.01	0.39	0.03	0.48	2.10	0.84	12

Data from 13 sugarcane trials including treatments with straw left on soil

AN, Ammonium nitrate; CAN, Calcium ammonium nitrate; CN, Calcium nitrate, AS, Ammonium sulphate; UR, Urea; UR + DCD, Urea + Dicyandiamide nitrification inhibitor; UR + DMPP, Urea + Dimethylpyrazole Phosphate nitrification inhibitor; PSCU, polymer sulphur-coated urea. SD, standard deviations; SEM, Standard error of the mean, IQR, interquartile range; n, number of observations

*Combined or not with vinasse

### Cross-wavelet analysis on emissions time series data

**Fig. 2:**
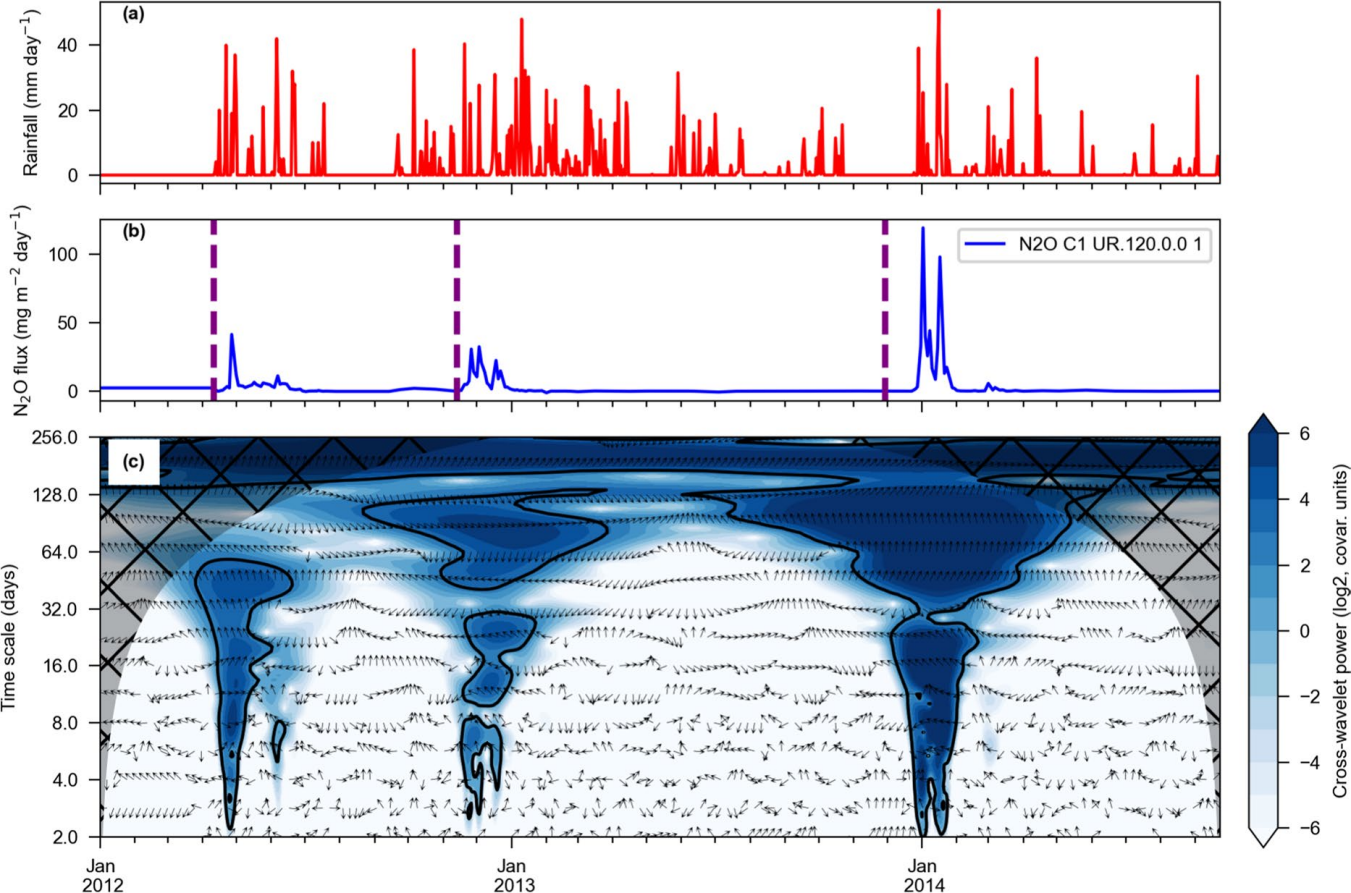
Cross-wavelet analysis between rainfall (**a**) and N_2_O flux (**b**) measured at trial C1 from 2012 to 2014. Cross-wavelet power (**c**) on log2 scale with units proportional to the covariance between the two signals. The area strongly influenced by the signals' edges is not considered and marked with a hatched pattern. The black contours enclose regions where the cross-wavelet power is statistically significant against a red noise background. Arrow angles represent the phase between the signals (clockwise reference). Fertiliser application dates marked with vertical dashed lines

Cross-wavelet analyses were carried out between N_2_O fluxes and two variables: rainfall and air temperature (Fig. 2 and Figure S4, respectively). The examples presented refer to the 2012–2014 trial number 1, with application of UR at the rate of 120 kg N ha^−1^. Examples were chosen as representatives of the general pattern observed in most of the experiments. Cross wavelet power, which is proportional to the covariance between rainfall and N_2_O flux (Fig. 2), is shown in the bottom panel in a logarithmic colour scale; in this panel, the arc-shaped shading denotes areas with low confidence due to edge effects near time series start and end. The black contours enclose regions where the cross-wavelet power is significantly different from a red noise background. Here a red noise is defined as a signal with a spectral energy density proportional to the reciprocal of the frequency squared. Finally, the arrows’ angle—in clockwise direction—denote the phase difference between peaks in the time series; upwards arrows (zero degrees) indicate perfectly aligned peaks, while right pointing arrows (90 degrees) indicate that rainfall leads the N_2_O flux, i.e., peaks in the rainfall series precede fluxes with lead times depending on the time scale on the vertical axis. Lead time τ is calculated as τ = (θ/360)P, where θ is the arrow angle, in degrees, and P is the cross-wavelet period. For example, the first event with significant cross-wavelet power in Fig. 2 occurred in April 2012, between time scale P of 2 to 64 days. The phase arrows point mostly upwards, indicating that rainfall peaks and N_2_O fluxes were mostly in sync in the monthly scale. The same patterns of upward arrows were observed for the experiment in 2014, up to the time scale of 64 days. However, short-lived peaks of N_2_O were observed to be delayed by 2–4 days in relation to rainfall events in April 2012. During that period, the cross-wavelet power is highest (colour scale) around the time scale of 8 days, which is consistent with the N_2_O’s peak width; the arrows in this region of the cross-wavelet panel are tilted around 90°–120°, which would result in N_2_O peaks with delays of 2–3 days after the rainfall. Lags were not observed during the periods in November 2012 or January 2014. However, according to the data records, the peak of 119.3 mg N m^−2^ day^−1^ on January 2nd, 2014 was preceded by a 39 mm in rainfall on December 29th, 2013; the subsequent peak of 98.1 mg N m^−2^ day^−1^ on January 17th, 2014 occurred after a rainfall event of 37.3 mm on January 15th, 2014. These lags are not evident in the cross-wavelet panel due to the limitations in the temporal resolution of chamber measurements, which took place every 3 days.

Peak N_2_O fluxes were significantly correlated with daily mean temperature at various time scales, with no clear pattern of temporal delays (Figure S4). This result suggests that N_2_O fluxes might be triggered after a threshold in air temperature, depending on N availability in soil, rainfall events and soil moisture conditions.

### Classification and regression tree analysis for daily emissions

**Fig. 3:**
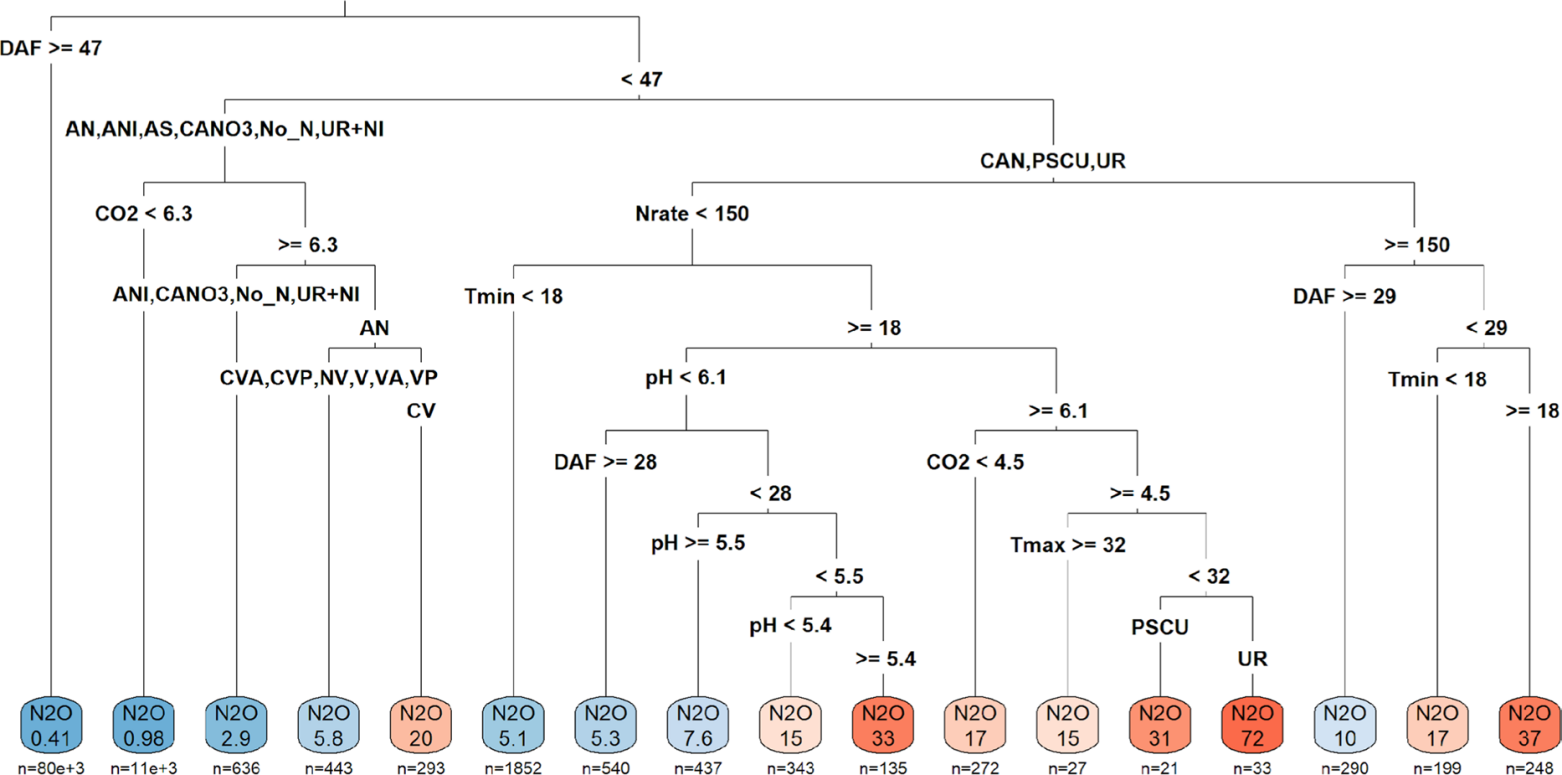
Regression tree (RT) relationship of N_2_O fluxes (mg N m^−2^ day^−1^) with management, climate, and soil conditions in the 13 sugarcane trials. The colour code in the figure ranges from blue (low emissions) to red (high emissions). DAF: days after fertiliser application (days); Fert_Rate: rate of N fertiliser (kg N ha^−1^); Tmin/Tmax: minimum/maximum temperature (°C); CO_2_ fluxes (g C m^−2^ day^−1^); OM: soil organic matter (mg kg^−1^); Prec: precipitation (mm/day); BD: Bulk density; P: phosphorus content in soil (mg P dm^−3^); n = number of observations

The RT analysis indicated that high N_2_O fluxes were distinguished from low values according to management, climate, and soil conditions (Fig. 3). The driver hierarchy in the RT model included DAF, fertiliser -N rate, N source, T_min_, pH, CO_2_, Vinasse, and T_max_. The first partitioning was due to DAF, in which low N_2_O-N fluxes are expected to occur when DAF > 47 days, independent of other conditions. The model predicted N_2_O-N emission of 0.41 mg N m^−2^ day^−1^ in DAF > 47 (Fig. 3). Low N_2_O emissions (< 1.0 mg N m^−2^ day^−1^) were also observed with DAF < 47, without N addition or with the N sources AN, AS, CN, or UR + inhibitors, and CO_2_ flux < 6.3 g C m^−2^. On the other hand, the highest N_2_O-N fluxes estimated were 37 mg N m^−2^ day^−1^, related to N sources CAN, PSC, or UR, in DAF < 29 days, N rate > 150 kg N ha^−1^ and T_min_ > 18 °C (Fig. 3). High fluxes were also correlated following: DAF < 47 days CO_2_ > 6.3 g C m^−2^, and the N source AN with CV; DAF < 47, N source CAN, PSC, or UR, N rate < 150 kg N ha^−1^, T_min_ > 18 °C, and pH 5.4–6.1; and DAF < 47, N rate < 150 kg N ha^−1^, T_min_ > 18 °C, pH > 6.1, CO_2_ > 4.5 g C m^−2^, T_max_ < 32 °C, with UR or PSC (Fig. 3).

### Correlation and regression analysis with a reduced database

**Fig. 4:**
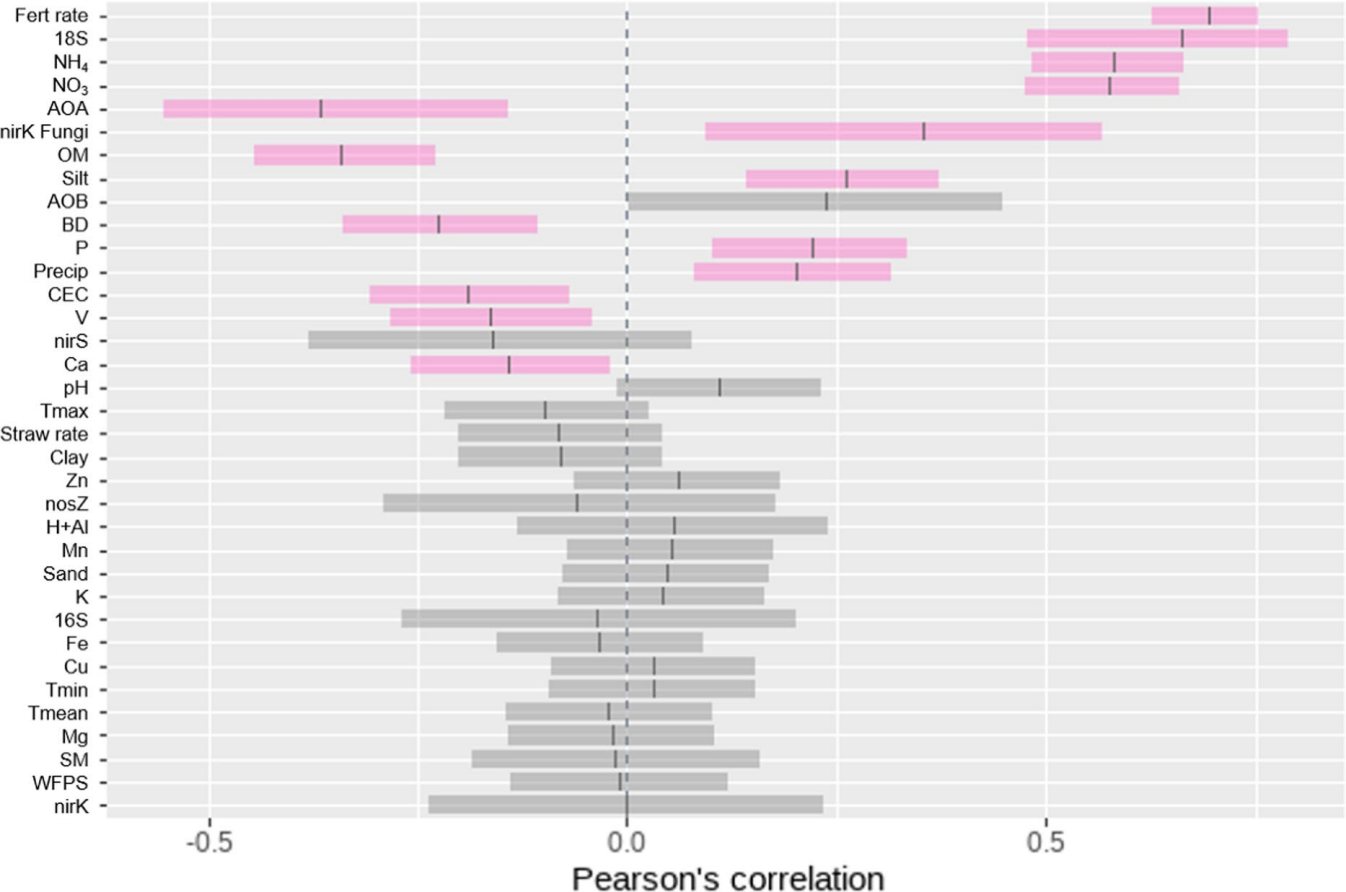
Pearson correlation coefficients between the cumulative N_2_O emissions in 46 days with soil, climate, and management variables. Pink colour means significant and grey means not significant at *p* < 0.05

Cumulative N_2_O emission, in 46 days resulted in positive and negative correlations with key soil, climate and management variables (Fig. 4). The highest correlation was found between N_2_O and N fertiliser rate, with a coefficient r = 0.69 (*p* < 0.001). Significant positive correlations with N_2_O were found for 18S rRNA gene (r = 0.66, *p* < 0.001), NH_4_^+^ (r = 0.58, *p* < 0.001), NO_3_^−^ (r = 0.57, *p* < 0.001), nirk fungi gene (r = 0.35, *p* < 0.01), silt content (r = 0.26, *p* < 0.001), soil P (r = 0.22, *p* < 0.001), and total precipitation in the period (r = 0.20, *p* < 0.01). Significant negative correlations were observed with N_2_O and archaeal amoA (r =  − 0.37, *p* < 0.01), soil organic matter (r =  − 0.34, *p* < 0.001), bulk density (r =  − 0.23, *p* < 0.001), CEC (r =  − 0.19, *p* < 0.01), V (r =  − 0.16, *p* < 0.01), and Ca (− 0.14, *p* = 0.02). No significant correlations were identified for other variables, including average maximum and minimum air temperature, despite apparent trends for higher emissions with higher temperature values (Figure S5).

According to multiple linear regression fits, the full set of variables including nitrogen fertiliser rate, straw rate, total precipitation, average maximum and minimum air temperature, and soil macro- and micro-nutrients, texture, CEC and WFPS explained ~ 65% of the cumulative N_2_O emission, suggesting that unmeasured biotic or abiotic factors explained the remaining 35% of the variation (Table 4). When the set of variables was reduced to include only parameters easily obtained by farmers such as fertiliser rate, the amount of straw left on the field after harvest, rainfall, and temperature on site, the regression still explained over 50% of the variation.

**Table 4:** Linear regressions for the influence of climate, soil and management parameters on cumulative N_2_O emissions in the first 46 days on reduced and complete sets of variables

Response	N_2_O*	N_2_O*
	Full set	Reduced set
*Coefficients*		
Intercept	− 87,270	1.7680661
Fert Rate	0.01458^+++^	0.0137162^+++^
Straw Rate	0.02508	− 0.062238^+++^
Prec	0.002276	0.0064778^+++^
T max	− 0.1922^+^	0.1625894^++^
T min	0.3674^+++^	− 0.2396474^+++^
CEC	0.1642^+^	
Mg	− 0.3114	
OM	− 1.212	
pH	0.9369^+++^	
V	0.009988	
Silt	− 0.000000002^+++^	
K*	9.349	
Ca*	252,800	
NH_4_*	0.07342	
NO_3_*	− 0.1796	
WFPS	0.02297^+^	
*Regression fit statistics*		
Multiple R^2^	0.68	0.53
Adjusted R^2^	0.65	0.52

^+++ ^, ^++^, ^+^ and . indicate coefficients significantly different to zero at *p* = 0.001, 0.01,0.05 and 0.1 levels, respectively. (*) denotes Box-Cox transformed data

## Discussion

### Fertiliser type significantly influenced emission factors and GHG intensity

Mean emission factors ranged from 0.03 to 1.17% of N applied over the thirteen experiment-years. These results combined the main management practices applied to sugarcane in Brazil and are in line with individual studies published (Carmo et al. 2013; Paredes et al. 2014; Soares et al. 2015; Siqueira Neto et al. 2016; Silva et al. 2017; Pitombo et al. 2017; Gonzaga et al. 2018; Borges et al. 2019; Lourenço et al. 2019; Degaspari et al. 2020; Cabral et al. 2020; Vasconcelos et al. 2022). In the present study, the mean values for N_2_O-EF, excluding nitrification inhibitor treatments, was 0.6%, which is lower than mean values previously reported, such as 0.7% for sugarcane Brazil (Carvalho et al. 2021), 1.2% for global sugarcane (Yang et al. 2021), and the default value of 1.6% from IPCC Tier 1 for regions with annual rainfall > 1000 mm (IPCC 2019). The N_2_O-EF depends on site-specific conditions, such as management, soil, and climate. In this way, a more detailed prediction as models (Tier 3) can better estimate N_2_O-EF (IPCC 2019) and the sustainability of the agricultural product.

Summarising N_2_O-EF by N sources, different values were found; using ammonium-based fertilisers, the mean N_2_O-EF was 0.5–0.6% (AN and CAN), while using UR-based sources it was 1% of N applied, but other N sources had N_2_O-EF lower than 0.1%. The range of N_2_O-EF occurred due to different management, e.g., the highest mean value for a treatment (3.15%) was observed when AN at 100 kg N ha^−1^ was combined with concentrated vinasse. Other high values of N_2_O-EFs included UR or CAN applied at a high N rate (180 kg ha^−1^) in soil covered with straw. In a meta-analysis study of N_2_O emission in sugarcane, Yang et al. (2021) observed higher emissions when combining synthetic fertiliser with organic amendments (N_2_O-EF: 2.47%) and increasing N rate application. On the other hand, low N_2_O-EFs (< 0.10%) were found in the present study, with CN, AN, and AS, as well as in the mitigation options: nitrification inhibitors addition in UR; and anticipated/postponed vinasse combined with AN. Therefore, the N management in sugarcane can show low N_2_O-EF options, which can be a strategy to mitigate GHG emissions (Carvalho et al. 2021). N_2_O emission intensity, represented as the cumulative N_2_O emissions in a growing season normalised by sugarcane yield, followed similar patterns to emission factors, with UR intensity generally higher than other fertiliser types. Fertiliser rates influenced N_2_O intensity, indicating that the increase in GHG emissions from excessive N application is not compensated by proportional increases in yield, as described by Takeda et al (2021) in a study monitoring emissions in a sugarcane plantation in Australia. Additionally, the emissions intensity of concentrated vinasse applied concomitantly with N fertiliser was significantly higher than emission intensity when vinasse was applied either 30 days before or 30 days after fertiliser, demonstrating the synergistic effect of combining organic amendments and mineral fertilisers on N_2_O emissions. When vinasse, an organic amendment with high moisture content, is applied along with fertiliser, a combination of increased soil moisture and corresponding increase in water filled pore space; and more nitrogen and labile carbon available for the soil microbial community can lead to increased soil N_2_O emissions, without a corresponding increase in sugarcane yields.

### N_2_O emissions were related to days after fertiliser application

When compared to the other variables studied, N management had the greatest impact on daily N_2_O flux. The high N_2_O fluxes occurred close to the time of N application, DAF < 47 days (Fig. 2); in addition, the N rate showed the highest correlation with cumulative N_2_O emission (Fig. 3) and was included in the OLS regression (Table 4). Increasing the N rate reduces nitrogen use efficiency in sugarcane (Chalco Vera et al. 2022; Sanches and Otto 2022), and increases N_2_O emissions, potentially priming organic N mineralization from soil (Degaspari et al. 2020; Takeda et al. 2022). In a study in Australia, Takeda et al. (2021) report that increasing fertiliser application beyond the recommended rate of 200 kg N ha^−1^ led to doubling the amount of N_2_O emitted per kg sugar yield—indicating an exponential effect.

The amount of straw left on the field did not have significant correlation with cumulative N_2_O emissions and was only significant in the linear regression with the reduced set of variables. The effect of straw on N_2_O emissions has been shown to have contrasting results as positive (Gonzaga et al. 2018) and negative effects (Pitombo et al. 2017). In a recent meta-analysis study, Abalos et al. (2022) reported an increase in N_2_O emissions from fresh crop residues (cover crops, grasslands and vegetables) but not from partially decomposed residues, with sugarcane postharvest straw showing no effect. Sugarcane straw can display diverse decomposition stages linked to accumulated residues from previous harvests, affecting soil microbial community and N_2_O emissions (Galdos et al 2010; Pitombo et al. 2017; Gonzaga et al. 2018).

### Environmental controls of N_2_O emissions

In the period close to fertiliser application, our analysis showed the temporal relationship (wavelet) impact of rainfall and air temperature on N_2_O fluxes. Zeri et al. (2020) showed that air temperature precedes the N_2_O peak by 10–20 days for biofuel crops (maize, miscanthus, switchgrass, and prairie). Here we found that the time lag between rainfall and N2O emission in sugarcane fields was 2 to 4 days on average during the first experiment in 2012, but not evident in the remaining measurements. According to Fig. 2b, N_2_O emissions were observed weeks after fertiliser application dates. The trigger is most likely soil moisture conditions (WFPS) reaching a threshold that enhances N_2_O production. The relationship between WFPS of 70% as a trigger to N_2_O emissions was reported by Metay et al. (2011), using simulations, and by Liang et al. (2018) over grazed pastures, which also associated the emissions to moderate soil temperature. The delays observed in April 2012 are most likely related to specific soil conditions at the time, or rainfall intensity, resulting in different soil wetting processes.

Soil moisture and temperature are the critical factors affecting microbial activity and diversity (Paul and Clark 1989). In dry conditions, soil moisture is more important than temperature, but when moisture is not limited, temperature is the major factor (Paul and Clark 1989). In general, higher production of N_2_O emissions in soil is expected to occur in WFPS between 50 and 75%, which is a more favourable condition for both nitrification and denitrification processes (Del Grosso et al. 2002; Liu et al. 2007). Increasing the temperature increases the rates of nitrification (Di and Cameron 2004) and denitrification processes (Braker et al. 2010), increasing N_2_O emissions. Chalco Vera et al. (2020) reported high N_2_O emissions increasing soil temperature (> 19 °C) and soil moisture (> 29.2%). However, Vargas et al. (2019) found that N_2_O emissions were higher in soil with temperature at 20 °C than at 30 °C. The study was conducted in controlled conditions, where the straw with a high C/N ratio probably causes a higher N immobilisation at higher temperatures. In the present study, over thirteen experiment-years of observation in the field, the N was applied in a band in soil (1.5 m row spacing), which elevates the soil N concentration and probably reduces the immobilisation effect compared with the broadcast-basis application of Vargas et al. (2019).

The delays found between N_2_O fluxes with rainfall and air temperature in cross wavelet analysis help to explain the low correlation (Pearson, r < 0.1) found between fluxes and both precipitation and air temperature in the linear regression analysis. Two sinusoidal curves which are completely in phase would result in a perfect correlation (r = 1) since peaks and valleys are aligned. Conversely, a phase difference, or delay, of 180 rad degrees would result in an inverse correlation (r =  − 1). However, in-between delays such as 90 degrees result in misaligned peaks and valleys, and consequent zero correlation. The N_2_O fluxes are a result of microbial processes in the soil, which are dependent on substrate availability (N), in addition to ideal conditions of soil temperature and moisture. Rainfall events were frequent during the occurrence of the N_2_O peaks, most likely creating ideal conditions of soil moisture for microbial activity and N_2_O fluxes. It should be noted that soil moisture and soil temperature were not available in all GHG sampling points in the trials, and rainfall and air temperature were used as proxies to represent thermal and moisture conditions in the soil.revious results on cross-wavelet analysis of N_2_O fluxes found no significant differences in delays when using air temperature or soil temperature (Zeri et al. 2020).

### Fungi abundance had a positive correlation with N_2_O emissions

Several factors had an impact on N_2_O fluxes, with strong positive correlations with fertiliser application rates, soil NH_4_^+^ and NO_3_^−^ content, and 18S rRNA genes, weak positive correlations with nirk fungi genes; and weak negative correlations with genes archaeal amoA (AOA) and soil organic matter content. The addition of organic C via vinasse and straw can result in an increase in microbial activity and in N_2_O emissions, where it could have more impact in soils with low OM (Lourenço et al. 2018). This effect can also explain the correlation of N_2_O with fungal activity (18S rRNA and nirk fungi genes), where the organic material supports favourable conditions for fungi growth, such as high moisture and organic C, leading to increases in N_2_O emissions (Lourenço et al. 2022). However, Yang et al. (2021) observed higher N_2_O emissions in soil with SOC > 20 g C dm^−3^ than with lower content; probably the organic C added in our study had a higher impact where soil C content was lower (11–16 g C dm^−3^), increasing N_2_O emissions. It is interesting that the relationship of N_2_O with bacterial amoA (AOB) was not significant, contrary to previous studies (Soares et al. 2016; Lourenço et al. 2018). The amoA gene codifies the nitrification process, which was the main process in individual studies, such as Soares et al. (2016) using UR in soil with no straw. Our results indicate that besides taking into account climate, management, and soil chemistry variables, soil microbiology should also be considered when monitoring N_2_O fluxes in sugarcane fields, a first step towards reducing emissions and reducing the carbon footprint of sugar and ethanol.

### Policy perspectives for GHG mitigation

Excessive input of reactive nitrogen (Nr) leads to high N losses posing significant threats to water, air, soil, biodiversity as well as GHG balance via direct and indirect emission of N_2_O (Galloway et al. 2004; Butterbach-Bahl et al. 2011). Aiming to meet the commitments of the Paris Agreement under the UNFCCC, the Brazilian government launched Law 13.576/2017, named “Renovabio”, which encourages the expansion of biofuel production to contribute to environmental preservation by reducing the emissions of GHG and other polluting gases (e.g., nitrogen oxides, particulate matter, etc.), in addition to promoting fuel security and economic and social development. The policy has been successfully adopted, as 90% of total ethanol plants in Brazil were certified in 2021 (Rossetto et al. 2022), motivated by the GHG reduction and economic benefits (Cantarella et al 2023).

Although sugarcane is considered the most suitable crop for bioenergy production worldwide (Miller 2010; Moraes and Zilberman 2014; Otto et al. 2022), its sustainability has been questioned due to its environmental and social impacts (Martinelli and Filoso 2008; Robinson et al. 2011; Immerzeel et al. 2014). Ways of reducing many negative impacts were addressed in the Renovabio legislation as bioenergy producers must comply with rules to benefit from decarbonization credits. For instance, bioenergy crops must not be grown in areas that have been deforested, even legally, after December 2017, crops must come from zoning areas allowed for specific feedstocks, and producers must abide by the Forest Code (Brasil 2017). In fact, sugarcane expansion occurred mostly in areas of pasture and cropland, where forest corresponded to less than 1% (Adami et al. 2012; Cherubin et al. 2021).

Problems mainly arise from the relatively low N-fertiliser recovery by sugarcane and the consequent high losses to the soil system (Chapman et al. 1994; Franco et al. 2011). Historically, Brazil has had a comparatively low input of N-fertiliser in sugarcane production (Baldani et al. 2002; de Matos Nogueira et al. 2005; Miller 2010). However, the expansion of sugarcane over the last decades increased the consumption of synthetic N-fertilisers. This is a complex situation, as fertiliser consumption is a critical indicator that determines the sustainability of nitrogen management in the country (Cunha-Zeri et al. 2022).

The present study identifies the main factors that affect N_2_O emissions in sugarcane production. An underlying factor is that as more fertiliser is used in sugarcane plantations, the risk of nitrogen being lost to the surrounding environment and causing pollution increases (Martinelli and Filoso 2008; Sutton et al. 2011). Therefore, monitoring in-field N_2_O fluxes is essential to ensure that the sustainability of sugarcane ethanol produced in Brazil remains favourable. Using IPCC tier 1 default values, Carvalho et al. (2021) estimated the cradle to wheel C footprint of ethanol and reported that ethanol reduced GHG emissions in 73% when replacing petrol; the avoided emissions could be increased by an additional 21% by adding nitrification inhibitors to N fertilisers used to produce sugarcane. Other policy responses should include strategies and investments in new technologies for sustainable agriculture, as well as a circular economy for nitrogen aimed at optimising the efficiency of resource use without waste and pollution (Sutton et al. 2019).

## Conclusions

The present study identifies important factors controlling N_2_O emissions in sugarcane fields in Southeastern Brazil. The N management had a high impact, where the high N_2_O fluxes occurred in the first 46 days after fertiliser application. There were also significant positive correlations between cumulative N_2_O and N rate, soil NH_4_^+^ and NO_3_^−^, and the genes 18S and fungal nirk; and negative correlations with organic matter and genes archaeal amoA. The mean N_2_O-EFs ranged from 0.03 to 1.17% of the N applied, with high emissions occurring with urea (UR), increasing N rate, and applying ammonium nitrate (AN) combined with vinasse, and low N_2_O-EF occurring with ammonium sulphate, AN, calcium nitrate, and mitigation alternatives (nitrification inhibitors and timing of vinasse application). The rainfall and air temperature had a high influence on the emissions, preceding the N_2_O fluxes by 2 to 4 days in some experiments and being generally well correlated overall. Understanding the factors influencing N_2_O emissions in sugarcane production, including fertiliser and organic amendment type, timing and rate, crop residue management, climate and soil variables can help farmers, extensionists, researchers and policy makers in the development and implementation of climate change mitigation strategies.

### Supplementary Information

Below is the link to the electronic supplementary material.

Supplementary file1 (DOCX 2240 kb)

Supplementary file2 (PNG 5771 kb)

Supplementary file3 (PNG 67 kb)

Supplementary file4 (PNG 930 kb)

Supplementary file5 (PNG 192 kb)

Supplementary file6 (PNG 982 kb)
