# Supplementary material for: Multi-experiment assessment of soil nitrous oxide emissions in sugarcane
Source: Nutr Cycl Agroecosyst. 2023 Oct 21;127(3):375–92. doi: 10.1007/s10705-023-10321-w (PMC10657304; doi:10.1007/s10705-023-10321-w)
Supplement: Supplementary file 1 — Supplementary file1 (DOCX 2240 kb) [file 10705_2023_10321_MOESM1_ESM.docx]

**Multi-experiment assessment of soil nitrous oxide emissions in sugarcane**

**Supplementary Material**

**
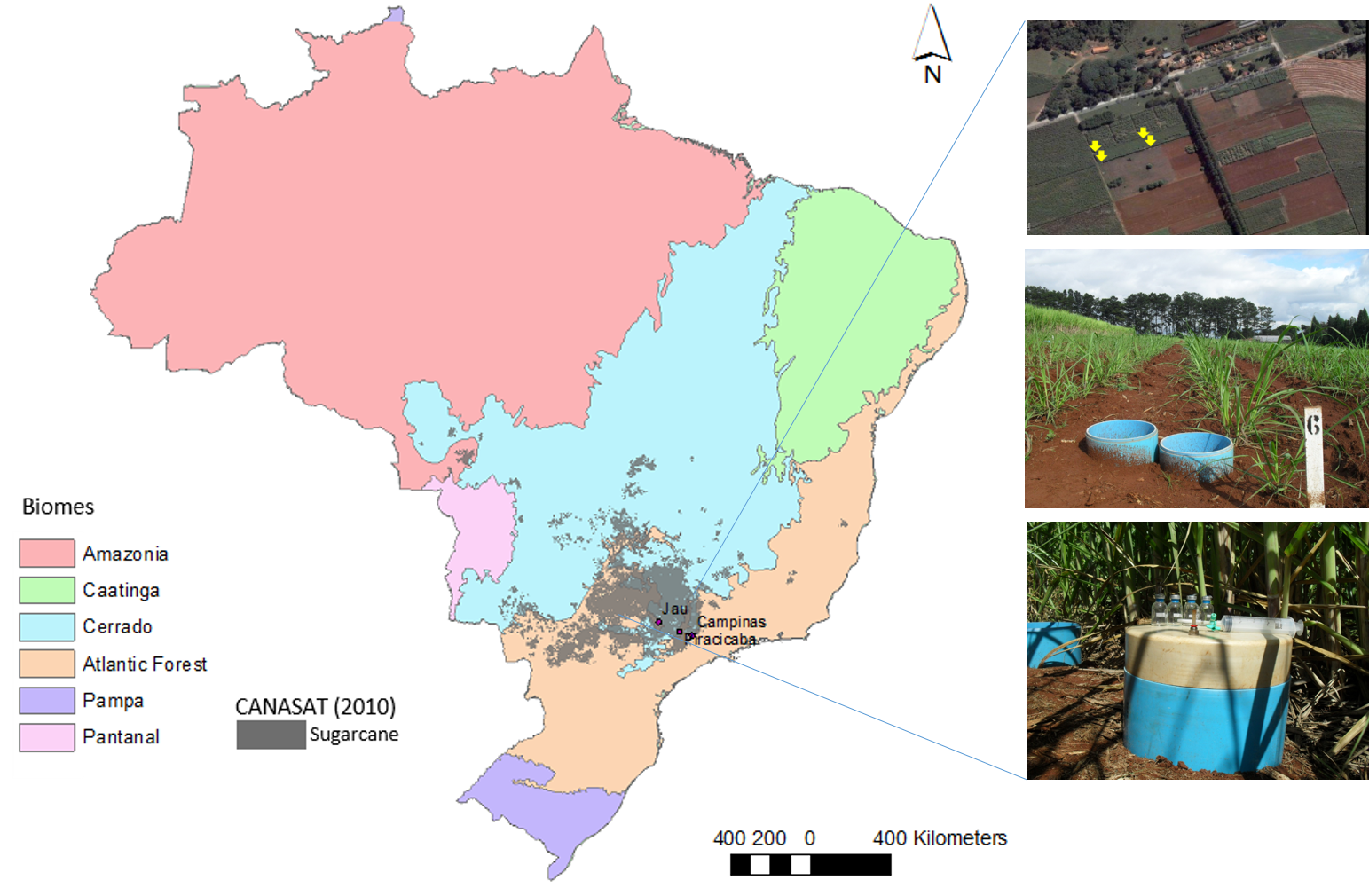
**

Figure S1. Location of the field experiments and sugarcane areas in Brazil with their biomes and with details of the experimental fields and chambers in the right panel.


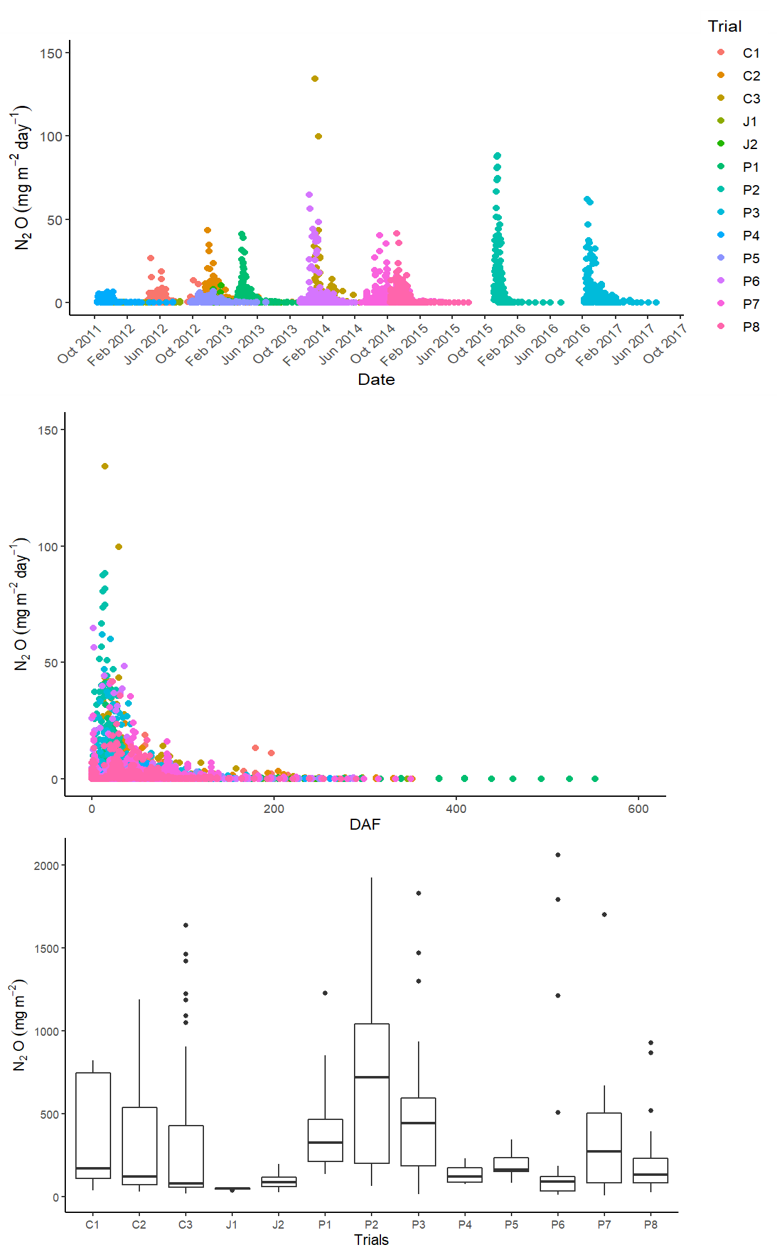


Figure S2. Daily N_2_O fluxes in the 13 sugarcane trials in the 2011-2017 period (top), in days after fertilizer application (middle), and as total N_2_O emissions in the season for each trial (bottom).

**Statistical Methods**

**Cross-wavelet analysis on emissions time series data**

The temporal relationships of N_2_O fluxes with mean daily rainfall and with air temperature were investigated using cross wavelet analysis, a variation of the wavelet transform applied to a pair of variables. The wavelet transform is a mathematical tool that enables the decomposition of a time series into its frequencies. Unlike the traditional Fourier transform, where contributions from each harmonic to the variance are averaged in a spectrum, the wavelet transform keeps time location in its decomposition. This feature is essential to describing the frequency of intermittent features in a time series. The wavelet transform has been used in several studies to identify non-periodic features in time series of river levels, turbulent fluxes over plant canopies, and variability of climate indices, among other applications (Gao and Li 1993; Sa et al. 1998; Torrence and Compo 1998; Yates et al. 2006, 2007; Furon et al. 2008; Zeri et al. 2020).

Temporal variability in wavelet analysis was quantified by the function:

W(t,a)= 1/√a ∫_(-∞)^∞ x(τ)〖Ψ〗^* ((τ-t)/a)dτ (2)

where t is the time and a is the parameter of scale used to stretch and shrink the “mother wavelet” Ψ, a function arbitrarily chosen to be convoluted with the time series; the * symbol denotes the complex conjugate; τ is the integration parameter used to displace the scaled wavelets along the time series x.

Mother wavelets are expected to follow some criteria, such as having zero mean and being localised in time (as opposed to infinite sine or cosine functions). Examples of typical functions used are the Morlet, Mexican hat, and Haar. The Morlet function was used in this study following previous applications in other studies (Furon et al. 2008; Zeri et al. 2020). This function is defined as:

Ψ(η)=〖π〗^(-1/4) 〖e〗^(i〖ω〗_0 η) 〖e〗^(-〖η〗^2/2) (3)

where non-dimensional parameters η and ω0 are the time and frequency, respectively.

The wavelet power, calculated as the squared absolute value of W(t,a), has units of the series variance. A variation of this tool for two variables is called the cross-wavelet, resulting in a wavelet power with units of covariance (Wxy). Cross-wavelet analysis quantifies the correlations in time and frequency between two variables (Grinsted et al. 2004). In addition, the complex part of Wxy has information about the phase difference between two time series, i.e., delays in time. A perfectly correlated series has zero delays between their peaks and troughs. Temporal shifts between peaks indicate that one process influences another later. For this study, the cross-wavelet analysis was conducted using scripts from the PyCWT package based on previous implementations of wavelet and cross-wavelet analysis (Torrence and Compo 1998; Grinsted et al. 2004).

**Classification and regression tree analysis for daily emissions**

Daily N_2_O fluxes were classified and correlated according to variables using a regression tree (RT) analysis (Breiman et al. 2017). The RT splits the data into subgroups (nodes), using a stepwise procedure until improvements are made in the model, resulting in the smallest tree. The pruned classification uses the Gin criterion for qualitative variables and the least squares regression for quantitative data, maximising homogeneity and reducing risk. Thereafter, the model is cross-validated (Breiman et al. 2017).

The climate, soil, and management variables included in the model were: N rate; vinasse application; straw rate; days after fertilizer application (DAF); air temperature (Tmin, Tmean, Tmax); precipitation; water-filled pore space (WFPS); soil bulk density (BD); organic matter (OM); pH; H + Al; Exchangeable Ca; K; Mg; resin-extracted P; DTPA-TEA extracted Cu, Fe, Mn, and Zn; cation-exchange capacity (CEC); NH_4_+; NO_3_-; CH_4_ flux; CO_2_ flux; clay, silt, and sand content; gene abundance (archaeal and bacterial amoA, nirK, nirS, nosZ; 16S; 18S; fungal nirk); and sugarcane stalk yield. The RT was performed using the ANOVA method. The RT analysis was conducted using the R packages “rpart” choosing the method of ANOVA (Therneau et al. 2022) and plotted with the “rpart.plot” package (Milborrow 2022). The RT was performed using . The model resulted in a tree with 16 splits, with complexity parameter (cp) of 0.01, and reducing relative error from 1.00 to 0.50 (0.04 to 0.02).

**Correlation and regression analysis with a reduced database**

Based on the outputs of the RT analysis, we generated a subset of the database containing data from the first 46 days after fertilizer application. This subset was used for further exploratory and statistical analysis. The use of data obtained for a longer period of observation may have blurred our capacity of evaluating the effects of variables on N_2_O emissions because since when soil N concentration becomes low (i.e., as crops take up N from fertilizer application, N is lost by leaching, NH3 volatilization, etc.), factors that otherwise affect N_2_O emissions may not be perceived. Cumulative N_2_O fluxes in each replicate, treatment and site were assessed for correlations with soil, climate, and management parameters. Except for rainfall, for which cumulative values for the period were used, total N_2_O emissions were assessed for correlations with mean values of each parameter. Pearson correlations were plotted following a ranking order of positive and negative correlations, and significant correlations at the 0.05 level were identified. The inspectdf R package (Rushworth and Wilkins 2021) was used for correlation analysis.

Using the same data subset, multivariate linear regressions were constructed considering the influence of two sets of variables on the cumulative N2O emissions: a complete set including climate, management, and soil parameters; and a reduced set with information more easily obtained by farmers, including climate variables, N fertilizer rates and the amount of straw left after harvest. The regression coefficients were estimated through OLS using mixed model functions in the R nlme package (Pinheiro et al., 2019).


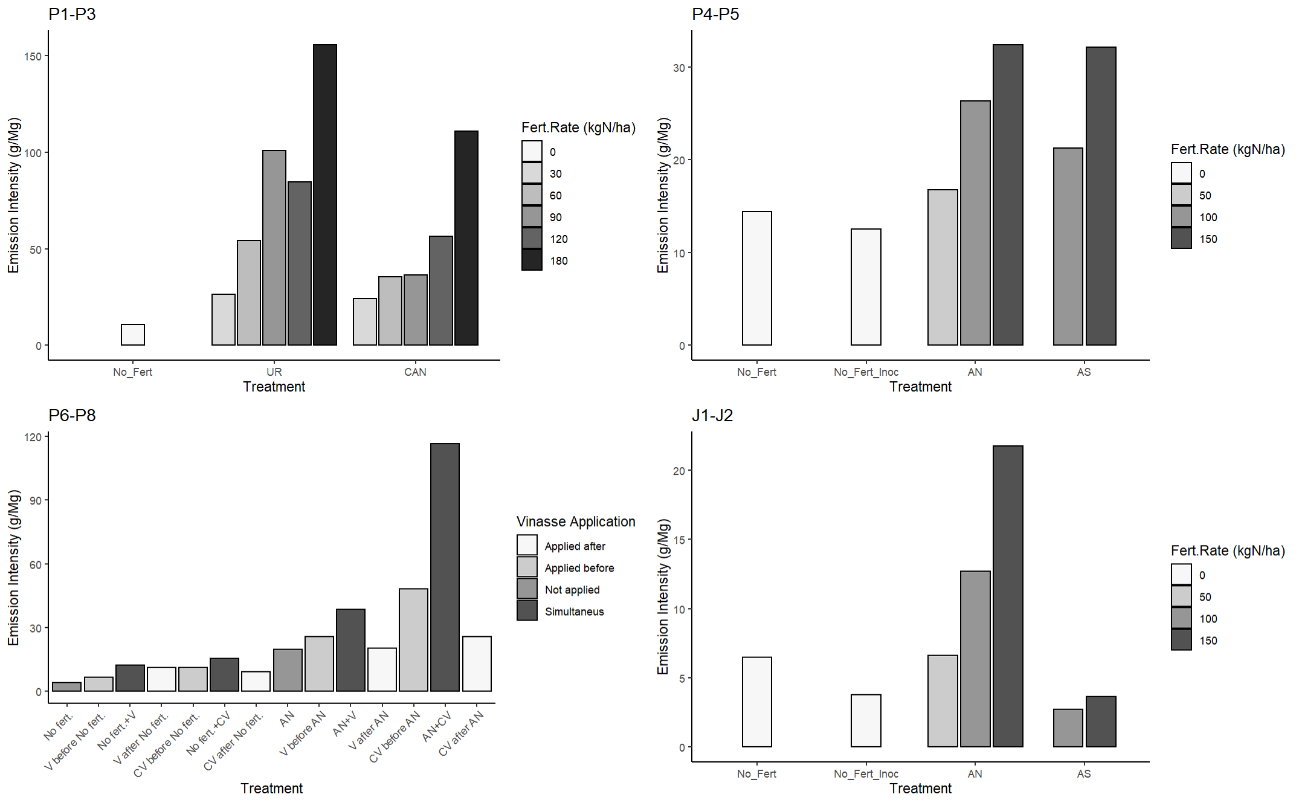


Figure S3. Nitrous oxide emission intensity per sugarcane stalk yield in the P1-3, P4-5, P6-8, and J1-2 trials, by fertilizer type followed by fertilizer rate in kg ha^-1^.


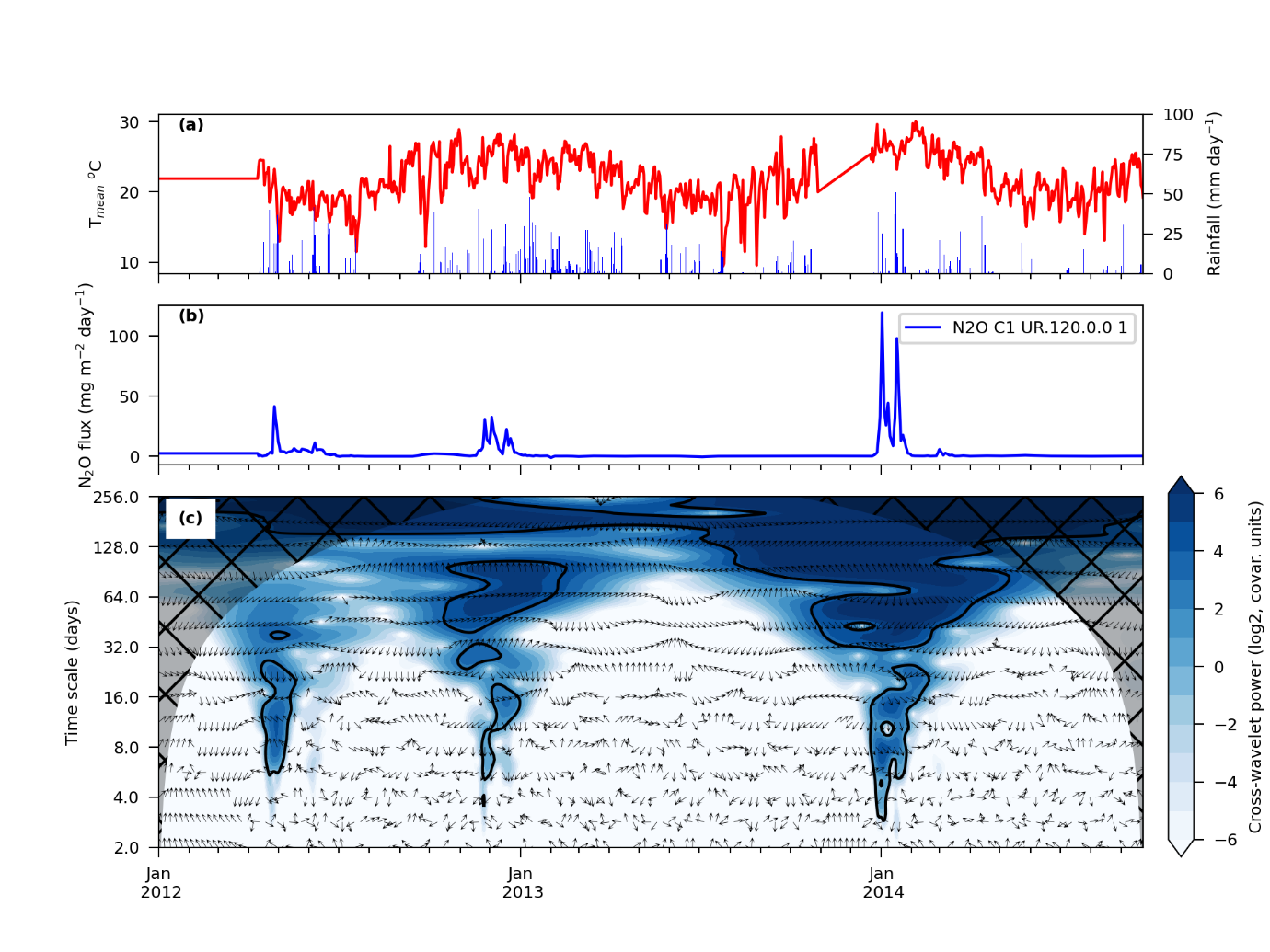


Figure S4. Cross-wavelet between mean daily air temperature (a) and N_2_O flux (b) measured at trial C1 from 2012 to 2014. Cross-wavelet power shown in panel (c) on log2 scale with units proportional to the covariance between the two signals. The area strongly influenced by the signals' edges is not considered and marked with a hatched pattern. The black contours enclose regions where the cross-wavelet power is statistically significant against a red noise background. Arrows angles represent the phase between the signals (clockwise reference).


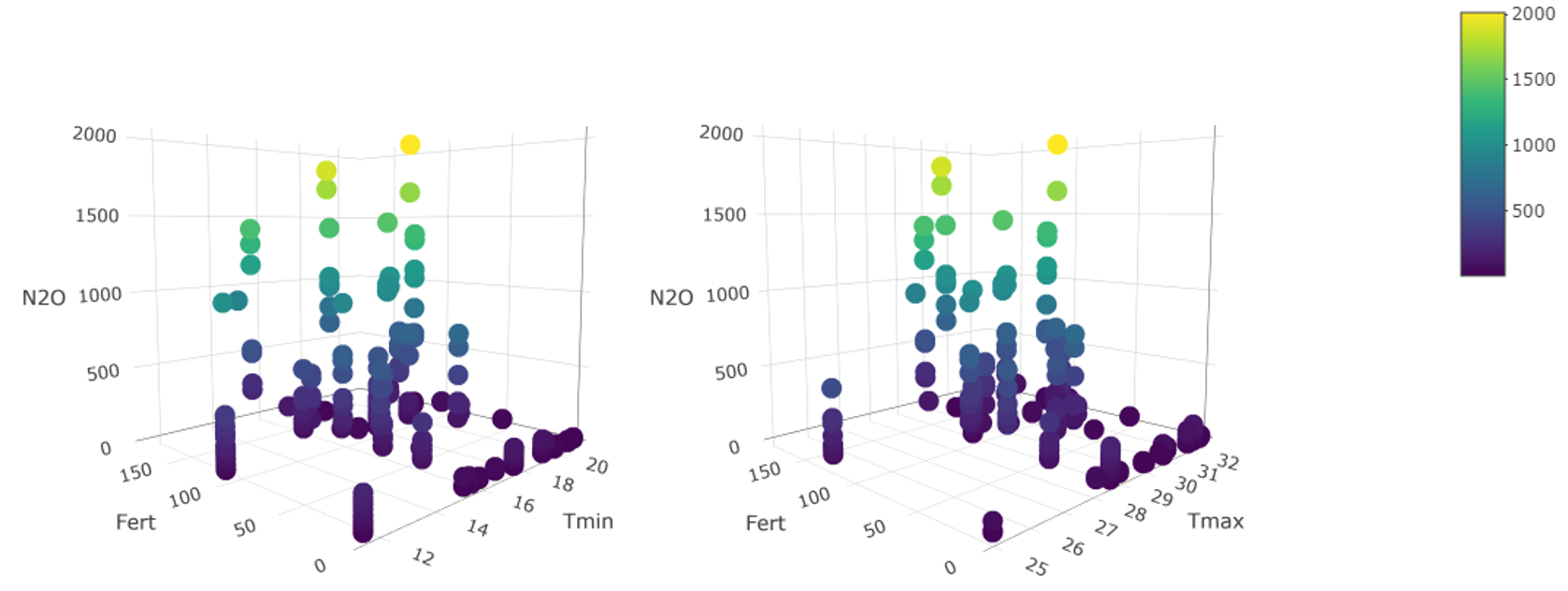


Figure S5. Cumulative N_2_O emissions in 46 days according to fertilizer application rate (kg N ha^-1^) and average daily minimum (a) and maximum (b) temperature.

Table S1. Summary statistics (p value) from ANOVA and pair-wise comparison tests (Tukey HSD, KW) for emission factors (EFs) conditional to conventional fertilizers and mitigation treatments in the trials (C1-C3, P1-P8, J1-J2).

| Trials/Treatments | ANOVA | Tukey HSD | KW |
| --- | --- | --- | --- |
| *P1-P3* | 0.000254 |  |  |
| UR-CAN |  | 0.00025 | 0.00044 |
| P4-P5 | 0.995 |  |  |
| AS-AN |  | 0.99498 | 0.56370 |
| *P6-P8* | 2.00E-16 |  |  |
| AN+CV-AN |  | 0.00000 | 1.74E-21 |
| AN+CV-AN+V |  | 0.00000 | 4.24E-06 |
| AN+CV-V after AN |  | 0.00000 | 3.77E-08 |
| AN+CV-V before AN |  | 0.00000 | 5.40E-10 |
| CV after AN - AN+CV |  | 0.00000 | 6.87E-06 |
| CV before AN - AN+CV |  | 0.00000 | 2.32E-01 |
| CV before AN-AN |  | 0.00919 | 3.87E-08 |
| AN+V-AN |  | 0.06967 | 2.71E-06 |
| CV before AN - V after AN |  | 0.13833 | 5.15E-04 |
| CV before AN - V before AN |  | 0.25758 | 4.30E-04 |
| CV after AN - CV before AN |  | 0.37292 | 1.14E-02 |
| V after AN-AN+V |  | 0.63322 | 3.62E-02 |
| CV before AN-AN+V |  | 0.77404 | 4.49E-02 |
| V before AN - AN |  | 0.84581 | 4.64E-02 |
| V before AN-AN+V |  | 0.89017 | 3.72E-02 |
| CV after AN-AN+V |  | 0.93772 | 2.66E-01 |
| CV after AN-AN |  | 0.95301 | 3.08E-02 |
| V after AN - V before AN |  | 0.99546 | 6.75E-01 |
| CV after AN - V after AN |  | 0.99880 | 3.99E-01 |
| V after AN-AN |  | 0.99971 | 2.40E-01 |
| CV after AN - V before AN |  | 1.00000 | 5.65E-01 |
| *C1-C3* | 2.00E-16 |  |  |
| PSCU-CANO3 |  | 0.00000 | 0.00034 |
| UR-CANO3 |  | 0.00000 | 0.00111 |
| UR+DCD-CANO3 |  | 0.98652 | 0.21654 |
| UR+DMPP-CANO3 |  | 1.00000 | 0.87164 |
| UR-PSCU |  | 0.63660 | 0.67306 |
| UR+DCD-PSCU |  | 0.00000 | 0.00022 |
| UR+DMPP-PSCU |  | 0.00000 | 0.00000 |
| UR+DCD-UR |  | 0.00000 | 0.00106 |
| UR+DMPP-UR |  | 0.00000 | 0.00000 |
| UR+DMPP-UR+DCD |  | 0.89998 | 0.05374 |
| *J1-J2* | 0.28450 |  |  |
| AS-AN |  | 0.28449 | 0.24820 |
|  |  |  |  |

UR: Urea; UR+DCD: Urea with Dicyandiamide nitrification inhibitor; UR+DMPP: Urea with 3,4-Dimethylpyrazole Phosphate nitrification inhibitor; PSCU: Polymer sulfur coated urea; CN: Calcium nitrate, CAN: Calcium ammonium nitrate, AS: Ammonium sulfate; AN: Ammonium nitrate: CV: Concentrated vinasse; *rate for concentrated vinasse; VN0: vinasse with no N fertilizer; V+N: vinasse applied with N; V/N: Vinasse applied 30 days before N; N/V: Vinasse applied 30 days after N.

Table S2. Summary statistics for emission factors (EFs) conditional to conventional fertilizers and mitigation treatments in the trials (C1-C3, P1-P8, J1-J2).

| Trials/Treatments | diff | lwr | upr | p adj |
| --- | --- | --- | --- | --- |
| *P1-P3* |  |  |  |  |
| UR-CAN | 0.39175 | 0.18908 | 0.59442 | 0.00025 |
| *P4-P5* |  |  |  |  |
| AS-AN | 0.00042 | -0.19496 | 0.19580 | 0.99498 |
| *P6-P8* |  |  |  |  |
| AN+V-AN | 0.25628 | -0.01094 | 0.52350 | 0.06967 |
| V before AN - AN | 0.13338 | -0.16868 | 0.43544 | 0.84581 |
| V after AN-AN | 0.04865 | -0.31968 | 0.41699 | 0.99971 |
| AN+CV-AN | 1.12473 | 0.85751 | 1.39195 | 0.00000 |
| CV before AN-AN | 0.43596 | 0.06763 | 0.80430 | 0.00919 |
| CV after AN-AN | 0.12431 | -0.24403 | 0.49264 | 0.95301 |
| V before AN-AN+V | -0.12291 | -0.42497 | 0.17916 | 0.89017 |
| V after AN-AN+V | -0.20763 | -0.57596 | 0.16071 | 0.63322 |
| AN+CV-AN+V | 0.86845 | 0.60123 | 1.13566 | 0.00000 |
| CV before AN-AN+V | 0.17968 | -0.18866 | 0.54801 | 0.77404 |
| CV after AN-AN+V | -0.13198 | -0.50031 | 0.23636 | 0.93772 |
| V after AN - V before AN | -0.08472 | -0.47907 | 0.30962 | 0.99546 |
| AN+CV-V before AN | 0.99135 | 0.68929 | 1.29341 | 0.00000 |
| CV before AN - V before AN | 0.30259 | -0.09176 | 0.69693 | 0.25758 |
| CV after AN - V before AN | -0.00907 | -0.40341 | 0.38527 | 1.00000 |
| AN+CV-V after AN | 1.07607 | 0.70774 | 1.44441 | 0.00000 |
| CV before AN - V after AN | 0.38731 | -0.05983 | 0.83445 | 0.13833 |
| CV after AN - V after AN | 0.07565 | -0.37149 | 0.52279 | 0.99880 |
| CV before AN - AN+CV | -0.68877 | -1.05710 | -0.32043 | 0.00000 |
| CV after AN - AN+CV | -1.00042 | -1.36876 | -0.63209 | 0.00000 |
| CV after AN - CV before AN | -0.31166 | -0.75880 | 0.13549 | 0.37292 |
| *C1-C3* |  |  |  |  |
| PSCU-CANO3 | 1.13648 | 0.69714 | 1.57583 | 0.00000 |
| UR-CANO3 | 0.98278 | 0.54344 | 1.42212 | 0.00000 |
| UR+DCD-CANO3 | 0.07505 | -0.34175 | 0.49185 | 0.98652 |
| UR+DMPP-CANO3 | -0.00112 | -0.41791 | 0.41568 | 1.00000 |
| UR-PSCU | -0.15370 | -0.46437 | 0.15696 | 0.63660 |
| UR+DCD-PSCU | -1.06144 | -1.33930 | -0.78357 | 0.00000 |
| UR+DMPP-PSCU | -1.13760 | -1.41547 | -0.85974 | 0.00000 |
| UR+DCD-UR | -0.90773 | -1.18560 | -0.62987 | 0.00000 |
| UR+DMPP-UR | -0.98390 | -1.26176 | -0.70603 | 0.00000 |
| UR+DMPP-UR+DCD | -0.07616 | -0.31680 | 0.16447 | 0.89998 |
| *J1-J2* |  |  |  |  |
| AS-AN | -0.08124 | -0.28015 | 0.11766 | 0.28449 |

UR: Urea; UR+DCD: Urea with Dicyandiamide nitrification inhibitor; UR+DMPP: Urea with 3,4-Dimethylpyrazole Phosphate nitrification inhibitor; PSCU: Polymer sulfur coated urea; CN: Calcium nitrate, CAN: Calcium ammonium nitrate, AS: Ammonium sulfate; AN: Ammonium nitrate: CV: Concentrated vinasse; *rate for concentrated vinasse; VN0: vinasse with no N fertilizer; V+N: vinasse applied with N; V/N: Vinasse applied 30 days before N; N/V: Vinasse applied 30 days after N.
