# Supplementary figures and images for: Multi-experiment assessment of soil nitrous oxide emissions in sugarcane
Source: Nutr Cycl Agroecosyst. 2023 Oct 21;127(3):375–92. doi: 10.1007/s10705-023-10321-w (PMC10657304; doi:10.1007/s10705-023-10321-w)

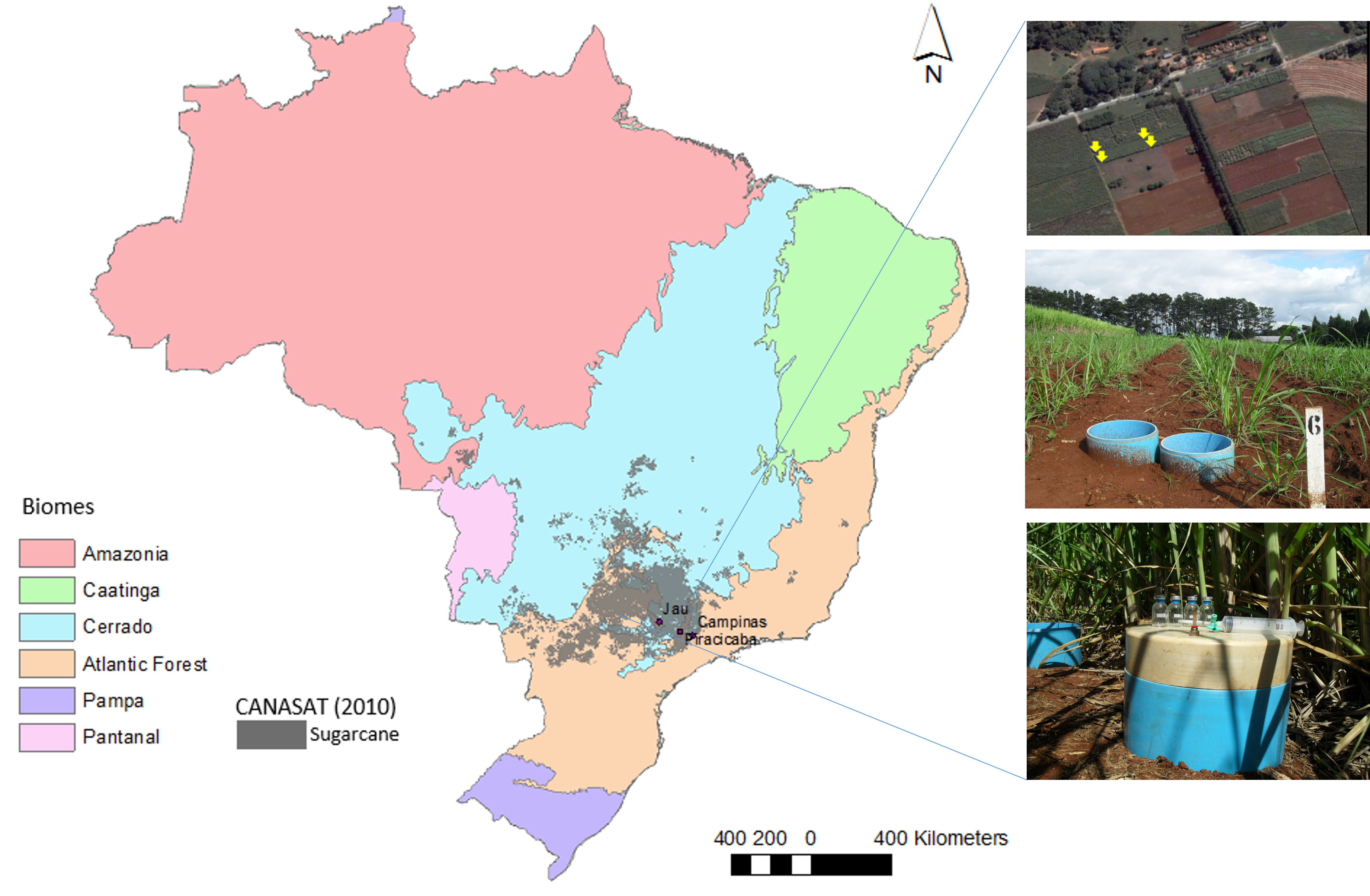

Supplement: Supplementary file 2 — Supplementary file2 (PNG 5771 kb) [file 10705_2023_10321_MOESM2_ESM.png]

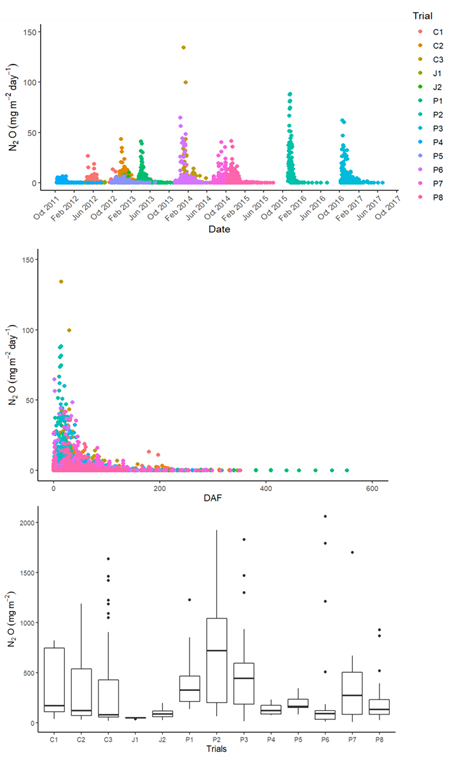

Supplement: Supplementary file 3 — Supplementary file3 (PNG 67 kb) [file 10705_2023_10321_MOESM3_ESM.png]

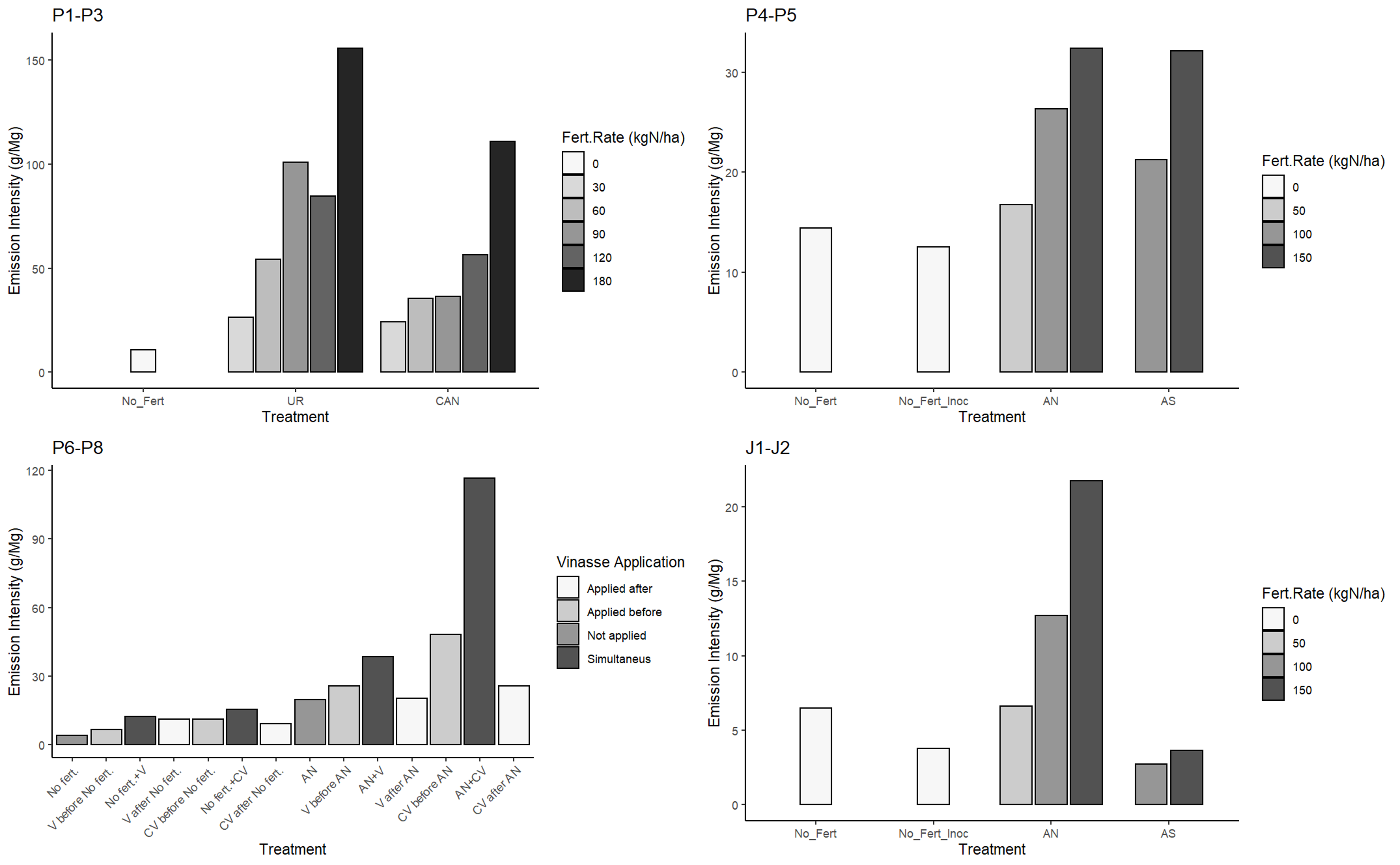

Supplement: Supplementary file 4 — Supplementary file4 (PNG 930 kb) [file 10705_2023_10321_MOESM4_ESM.png]

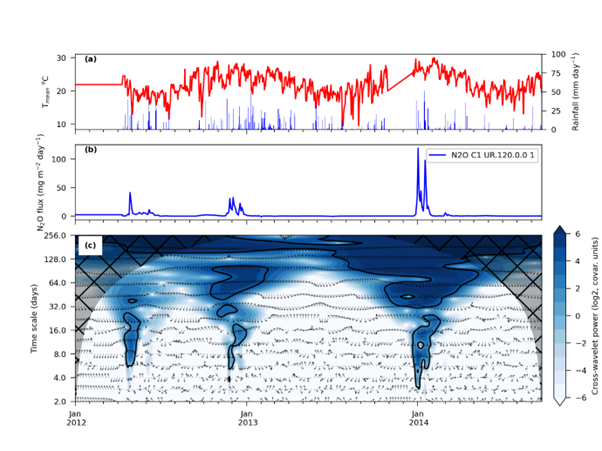

Supplement: Supplementary file 5 — Supplementary file5 (PNG 192 kb) [file 10705_2023_10321_MOESM5_ESM.png]

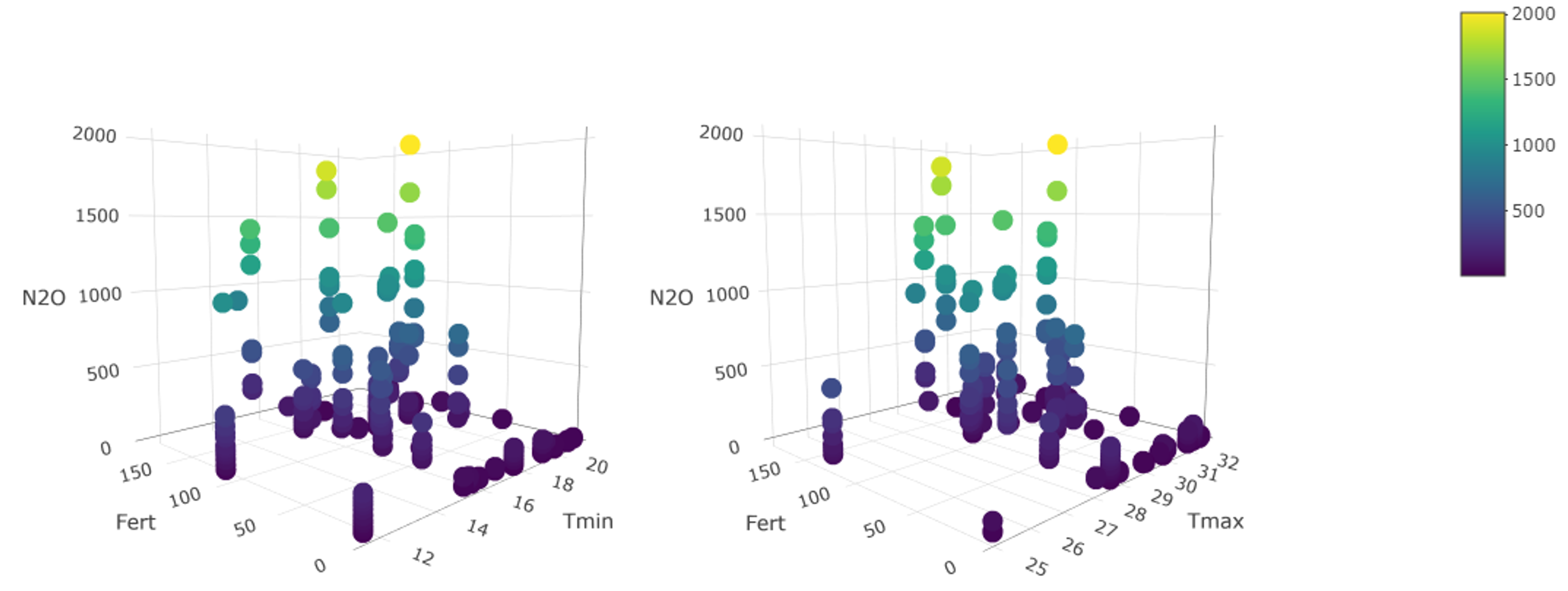

Supplement: Supplementary file 6 — Supplementary file6 (PNG 982 kb) [file 10705_2023_10321_MOESM6_ESM.png]
